# A stem cell-based assay platform demonstrates alpha-synuclein dependent synaptic dysfunction in patient-derived cortical neurons

**DOI:** 10.1038/s41531-024-00725-y

**Published:** 2024-05-21

**Authors:** Andrew J. White, Karis A. Clark, Kellianne D. Alexander, Nagendran Ramalingam, Tracy L. Young-Pearse, Ulf Dettmer, Dennis J. Selkoe, Gary P. H. Ho

**Affiliations:** Ann Romney Center for Neurologic Diseases, Department of Neurology, Brigham and Women’s Hospital and Harvard Medical School, Boston, MA 02115 USA

**Keywords:** Parkinson's disease, Cellular neuroscience

## Abstract

Alpha-synuclein (αS)-rich Lewy bodies and neurites in the cerebral cortex correlate with the presence of dementia in Parkinson disease (PD) and Dementia with Lewy bodies (DLB), but whether αS influences synaptic vesicle dynamics in human cortical neurons is unknown. Using a new iPSC-based assay platform for measuring synaptic vesicle cycling, we found that in human cortical glutamatergic neurons, increased αS from either transgenic expression or triplication of the endogenous locus in patient-derived neurons reduced synaptic vesicle cycling under both stimulated and spontaneous conditions. Thus, using a robust, easily adopted assay platform, we show for the first time αS-induced synaptic dysfunction in human cortical neurons, a key cellular substrate for PD dementia and DLB.

## Introduction

Dementia is a highly debilitating but poorly understood feature of PD and DLB^1^. The shared defining pathological feature of these disorders, the Lewy body, is comprised primarily of the abundant neuronal protein αS^2^. High levels of αS can inhibit various stages of the synaptic vesicle cycle, thus implicating it in the dementia phenotype^3^. In accord, duplication^4^ and triplication^5^ of the *SNCA* gene encoding αS cause familial PD/DLB^5^. To date, the specific effects of αS on synaptic vesicle dynamics have been studied exclusively in rodent neurons or adrenal chromaffin cells. In these systems, transgenically expressed human αS 1) disrupts re-clustering of synaptic vesicles, reducing the size of the recycling pool^6^; 2) restricts the mobility of vesicles within individual synapses^7^ and across multiple boutons^8^; and 3) impairs vesicle exocytosis^6,9,10^. This body of work has been instrumental to our understanding of αS synaptic biology. However, primary rodent neurons have limitations in terms of modeling human synucleinopathies. It is not possible, for instance, to account for all the incompletely known physiological differences between rat and human neurons when studying human neurodegenerative disease. In considering αS specifically, wild-type (wt) rat and mouse αS both contain a threonine at position 53, which corresponds to the αS-A53T missense mutation found in one form of autosomal dominantly inherited PD in humans^11^. Thus, wt rodent αS already contains a known pathogenic human αS mutation. In studies of αS in mouse or rat neurons, human αS-wt is typically ectopically expressed against this background^6,8,12^, thereby complicating the interpretation of results in a disease-relevant context.

In addition to species considerations, the neuronal type used to study synaptic effects of αS is important. The work in rodent neurons described above used adrenal chromaffin^9^, hippocampal^7,8^, or dopaminergic^6^ cultures. Synaptic activity has also been studied in human dopaminergic neurons by electrophysiology, without direct assessment of synaptic vesicle dynamics^13,14^. While the hippocampus and dopaminergic neuron-rich substantia nigra are key anatomical areas involved in PD, how pathological αS affects synaptic vesicle cycling in the neocortex is unknown. This is a critical question in the molecular mechanisms of synucleinopathy-associated dementia because the extent of Lewy body pathology in the neocortex correlates with the probability of having the dementia syndrome typical of PD dementia and DLB^15^.

**Fig. 1:**
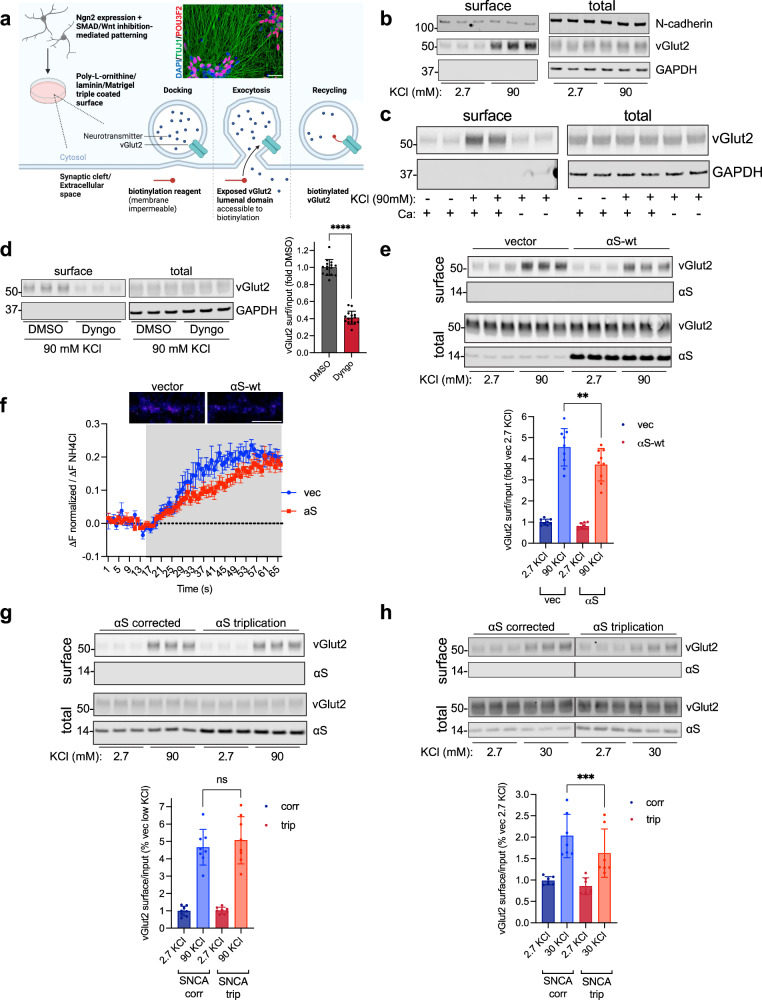
The surface biotinylation-based method specifically detects synaptic vesicle cycling in human iPSC-derived cortical neurons and demonstrates αS-dependent reduced evoked vesicle cycling. **a** Schematic of our assay platform. Inset, BR33 iNs on DIV21 were fixed and immunostained with neuronal marker TUJ1, and layer II/III cortical neuron marker POU3F2 (Brn2). Nuclei are stained with DAPI. Scale bar, 10 µm. **b** Biotinylation of constitutive surface proteins is unaffected by KCl-mediated depolarization. iNs were stimulated with 90 mM KCl for 60 s and subjected to surface biotinylation with maleimide-PEG2-biotin followed by pulldown, elution, and WB for the indicated proteins. **c** Activity-dependent surface biotinylation of vGlut2 requires calcium. iNs were stimulated with 90 mM KCl for 60 s in the presence or absence of 1.7 mM CaCl_2_ followed by surface biotinylation and WB as in **b**. **d** Surface biotinylation assay measures both exo- and endocytosis. Endocytosis was blocked with the dynamin inhibitor Dyngo (30 µM) followed by surface biotinylation with 90 mM KCl stimulation as in **b**. *N* = 15 biological replicates. Data are plotted as means ± SD, ****p < 0.0001, unpaired 2-tailed t-test. **e** Ectopic αS reduces synaptic vesicle cycling. *Top*, synaptic vesicle cycling in iNs stably expressing either αS-wt or vector control was analyzed by surface biotinylation with maleimide-PEG2-biotin. *Bottom*, quantification of the vGlut2 surface signal, normalized to total, expressed as a percentage of vector control. *N* = 9 biological replicates. Data are plotted as means ± SD, **p < 0.01, 1-way ANOVA with Sidak’s multiple comparisons test. **f** Synaptic vesicle cycling of the iNs in **e** was analyzed by an orthogonal method, live imaging of synaptopHluorin (sypHy) fluorescence with KCl stimulation. Shown is the average fluorescent sypHy signal (±SEM) over time, expressed as a % of the NH_4_Cl-responsive puncta. Gray shaded area indicates period of incubation with 90 mM KCl. *Inset*, pseudocolored representative sypHy fluorescent images of stimulated neurites. Scale bar, 5 µm. **g** Synaptic vesicle cycling in αS triplication vs isogenically corrected control was assessed by the surface biotinylation method as in **e**. *N* = 8 biological replicates. Data are plotted as means ± SD, ns, not significant, 1-way ANOVA with Sidak’s multiple comparisons test. **h** Synaptic vesicle cycling in αS triplication is reduced compared to isogenically corrected control with 30 mM KCl stimulation. Synaptic vesicle cycling was assessed as in **e** but with 30 mM KCl stimulation instead of 90 mM. *N* = 7 biological replicates. Data are plotted as means ± SD, ***p < 0.001, 1-way ANOVA with Sidak’s multiple comparisons test.

To address these two issues of species specificity and cortical cell type identity, we applied the neurogenin-2 based method of differentiating human induced pluripotent stem cells (iPSCs) into cortical glutamatergic neurons (iNs)^16^. They allow for investigations in a human cell background, exhibit characteristics of excitatory neurons of the layer II/III neocortex^16^, and become electrically active within 21 days of differentiation^17^. To measure synaptic cycling, we sought to do so with as little experimental manipulation of endogenous function as possible. The current convention, imaging of synaptic vesicle exocytosis with genetically overexpressed pH-sensitive GFP (pHluorin) based probes, requires the use of transgenes which could alter synaptic function. We thus became interested in a different quantitative method based on surface biotinylation of endogenous vesicle proteins. This method was described once previously in rat neurons^18^ but has not been used since in the reported literature, and its applicability to iPSC-derived neurons, which have different electrophysiological properties^19^, is unclear. In this method (Fig. 1a), cultures are bathed in extracellular solution (ECS) containing a membrane-impermeable surface biotinylation reagent. Endogenous vesicle proteins normally reside within the membrane of intracellular vesicles and are therefore inaccessible to biotinylation. Upon exocytosis and vesicle fusion with the presynaptic membrane, these proteins become exposed to the ECS and are biotinylated. Thus, the extent of surface biotinylation correlates with the amount of synaptic vesicle cycling^18^.

This method has properties complementary to the commonly used pHluorin-based techniques for assessing synaptic vesicle dynamics. pHluorin is fused to a synaptic vesicle protein such as synaptophysin, creating a reporter molecule, synaptopHluorin^20,21^. Since pHluorin only fluoresces at neutral pH, a signal occurs when vesicles are exocytosed and exposed to the neutral extracellular fluid pH, which then undergoes quenching with subsequent endocytosis and re-acidification. While the pHluorin approach has yielded valuable insights into presynaptic mechanisms, the surface biotinylation method offers certain advantages. First, it does not require expression of a transgenic reporter like synaptopHluorin. This is an important consideration because synaptopHluorin (and related reporters) are themselves functional synaptic vesicle proteins and their transgenic expression could alter synaptic function. Second, surface biotinylation is performed on an entire well of a culture plate, eliminating potential bias from region-of-interest selection in imaging-based techniques employing pHluorin. Third, the surface biotinylation method is facile to perform and does not require any special equipment, rendering it broadly adoptable by labs with differing expertise. In contrast, pHluorin-based assays require a substantial initial investment of equipment, including a fluorescent, (ideally confocal) microscope capable of live video recording, a perfusion system with pump and Transistor-Transistor-Logic (TTL) generator, as well as a specialized heated live imaging chamber for experiments at physiological temperature (a particularly relevant consideration when studying αS)^22^. A third method based on a similar principle as that of surface biotinylation is using a fluorescently tagged antibody against the luminal domain of synaptotagmin-1 to selectively visualize exocytosing vesicles^23^. In this case, labeling is based on an antibody binding interaction instead of covalent biotinylation, thus the resultant complex is substantially larger.

Here, we demonstrate the applicability of the surface biotinylation method for measuring synaptic cycling to human iNs in which we optimized cell viability and maturity with additional neuronal patterning and enhanced plate coatings. We developed the method into a rapid, tractable assay platform for studying αS-dependent effects on synaptic vesicle cycling and showed that iNs have robust and specific evoked and non-evoked (spontaneous) synaptic vesicle cycling. Over-expression of αS resulted in reduced evoked synaptic cycling induced by high (90 mM) KCl. Patient-derived endogenous αS triplication neurons also exhibited reduced evoked synaptic cycling, but only with less robust stimulation using 30 mM KCl. We then showed that our approach can also measure non-evoked, basal synaptic vesicle cycling. While electrophysiology has provided key insights into the role of αS in spontaneous rodent hippocampal synaptic activity^6,24,25^, this topic has not been studied in human cortical neurons using molecular methods such as ours. We found that both αS-transduced neurons and patient-derived endogenous αS triplication neurons exhibited reduced non-evoked synaptic cycling. Thus, our assay platform is a reproducible and easily adopted method for investigating presynaptic mechanisms affected by αS.

## Results and discussion

### The surface biotinylation method specifically detects synaptic vesicle cycling

Our initial steps were to optimize the neurogenin-2 trans-differentiated human iN system for measurement of synaptic activity. First, we incorporated extrinsic neuronal patterning with dual SMAD and Wnt inhibition during differentiation to promote synaptic maturity of the cultures^26^. Second, cells were plated on a triple coated surface consisting of poly L-ornithine, laminin, and Matrigel basement membrane matrix (instead of just one type of coating) to optimize viability and adherence. This triple coated surface was critical to cell adhesion during subsequent processing steps. Our control BR33 iNs from a non-PD, non-demented individual^17^ expressed neuronal markers specific to layer II/III cortical neurons, as expected (Fig. 1a, inset). In these cultures, we demonstrated the presence of synapses, as defined by co-localized puncta of the pre- and post-synaptic markers vGlut2 and Homer1, respectively (Supplementary Fig. 1). We then tested two types of membrane-impermeable biotinylation reagents, sulfo-NHS-SS-biotin and maleimide-PEG2-biotin. The NHS moiety is amine reactive and labels lysines in proteins, while the maleimide moiety is sulfhydryl reactive and labels cysteines. We selected vGlut2 as the endogenous vesicle protein to detect, as iNs are glutamatergic and express predominantly vGlut2 instead of vGlut1^16^. Both NHS-SS-biotin and maleimide-PEG2-biotin detected increased surface vGlut2 following high 90 mM KCl stimulation to induce exocytosis, with maleimide-PEG2-biotin producing a more robust signal (Supplementary Fig. 2). Surface labeling was specific, since actin, an intracellular protein, did not yield a signal. Thus in subsequent experiments we used maleimide-PEG2-biotin.

We then tested the specificity of monitoring surface vGlut2 to measure synaptic cycling activity by comparing it to other surface proteins not involved in synaptic vesicle dynamics. With 90 mM KCl stimulation, surface levels of vGlut2 were greatly increased as before (Fig. 1b). However, in the same experiments, surface levels of the surface adhesion marker N-cadherin remained unaltered by KCl stimulation (Fig. 1b). The plasma membrane calcium transporting ATPase-1 (PMCA1), a constitutive calcium transporter, was similarly unaffected (Supplementary Fig. 3). Furthermore, stimulating iNs in the absence of calcium in the medium, a vital signaling molecule in triggering synaptic vesicle fusion and exocytosis, prevented the 90 mM KCl-induced increase in surface vGlut2 (Fig. 1c).

To characterize how endocytosis influences our assay readouts, we measured surface vGlut2 following depolarization with 90 mM KCl in the presence or absence of the dynamin inhibitor Dyngo to block endocytosis^27^. Biotinylation of vGlut2 was significantly reduced with Dyngo (Fig. 1d), suggesting that the technique measures both exo- and endocytosis. These data also indicate that a single round of exocytosis is insufficient to completely label all vGlut2 molecules in the vesicle. If this were the case, then Dyngo treatment would not alter the amount of signal because either single or multiple rounds of endo- and exocytosis would produce equal and complete biotinylation of all vesicular vGlut2. Instead, with each successive synaptic vesicle cycle, more vGlut2 becomes biotinylated, perhaps due to incomplete accessibility to the maleimide-PEG2-biotin. The assay therefore measures total synaptic vesicle cycling over a given time interval. To confirm the dynamics of vesicle trafficking, we depolarized neurons with 90 mM KCl and then returned them to normal 2.7 mM KCl containing extracellular solution for 10 min. This resulted in a large reduction in surface vGlut2, consistent with endocytosis of synaptic vesicles after removal of the stimulus (Supplementary Fig. 4). Taken together, these results support the conclusion that surface biotinylation of vGlut2 using our method specifically measures dynamic synaptic events.

### αS over-expression and triplication of endogenous αS in patient-derived neurons inhibits stimulated synaptic vesicle cycling

We then sought to determine the effect of αS on synaptic vesicle cycling using our assay platform. We differentiated iNs stably over-expressing either αS or a vector control in the 2132 line background^28^ and subjected them to surface biotinylation with basal low (2.7 mM) and high (90 mM) KCl. Consistent with findings in rodent hippocampal neurons^6^ and adrenal chromaffin cells^9^, αS reduced stimulated synaptic vesicle cycling as measured by surface vGlut2 (Fig. 1e). To establish that our approach produces similar results to the more commonly used pHluorin method, we transduced these iNs with the synaptophysin-pHluorin (sypHy) fusion protein reporter and determined the change in synaptic fluorescence after KCl stimulation (Fig. 1f). This orthogonal technique showed a similar reduction in vesicle cycling (Fig. 1f), further supporting the validity of our approach. Next, we asked if analogous changes might occur in patient-derived neurons from individuals with familial forms of PD/DLB. For this, we used iNs derived from a patient with triplication of the endogenous SNCA gene encoding αS, which produces an aggressive, early-onset form of PD/DLB^29^. In contrast to the iNs expressing ectopic αS, we did not observe a significant decrease in stimulated (90 mM KCl) synaptic cycling in the patient-derived iNs compared to their isogenically corrected controls (Fig. 1g). We reasoned that this may be due to the highly potent stimulus of 90 mM KCl in the setting of a relatively modest (~2-fold) increase in the level of αS in the triplication iNs. Thus, we asked if a less potent stimulus might allow for detection of more subtle differences between the triplication and corrected iNs. In accord, with 30 mM KCl stimulation, αS triplication neurons exhibited reduced synaptic vesicle cycling compared to their isogenically corrected counterparts (Fig. 1h).

### αS over-expression and triplication of endogenous αS in patient-derived neurons inhibits spontaneous synaptic vesicle cycling

**Fig. 2:**
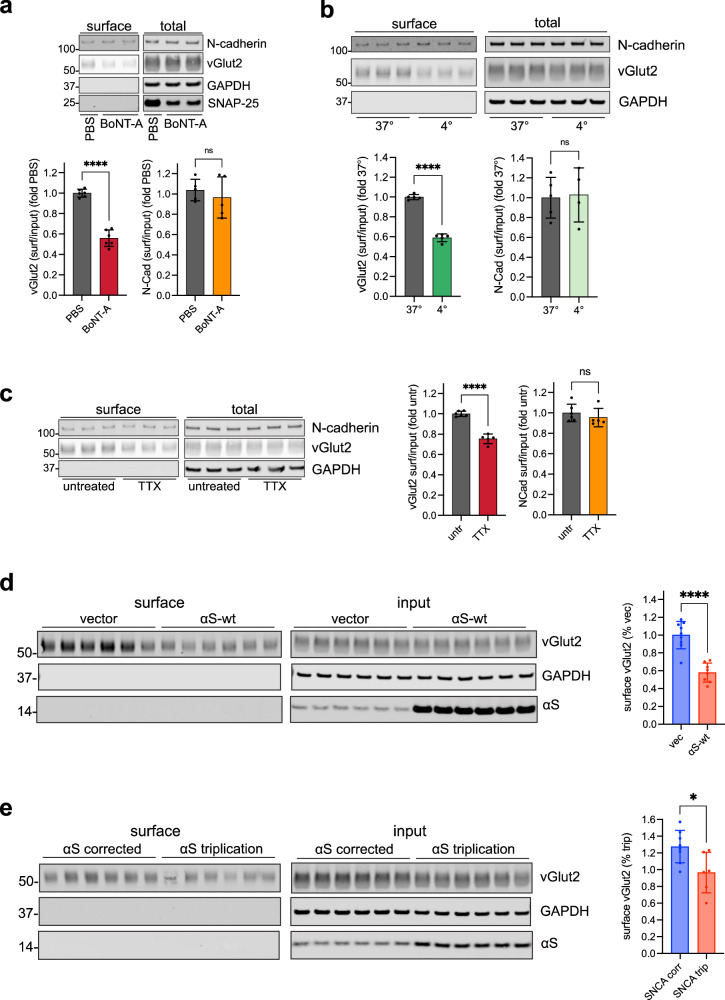
Non-evoked spontaneous synaptic cycling is reduced in αS-overexpressing and αS triplication patient-derived iNs. **a** Disruption of vesicle exocytosis under non-stimulated conditions reduces surface vGlut2 biotinylation. iNs were treated with 20 pM botulinum toxin subtype A complex and non-evoked synaptic cycling was measured by surface biotinylation. vGlut2 (bottom left) or N-cadherin (bottom right) surface signal was normalized to total and expressed as a percentage of the PBS vehicle control. *N* = 6 biological replicates. Data are plotted as means ± SD, ****p < 0.0001, ns not significant. Unpaired 2-tailed t-test. **b** Reducing synaptic vesicle cycling under non-stimulated conditions by lowering temperature reduces surface vGlut2 biotinylation. iNs were labeled under basal conditions at either 37 or 4 °C and normalized surface vGlut2 and N-cadherin measured as in **a**, expressed as a percentage of the 37° condition. *N* = 5 biological replicates. Data are plotted as means ± SD, ****p < 0.0001, ns, not significant. Unpaired 2-tailed t-test. **c** Surface biotinylation under non-evoked, spontaneous conditions captures both action potential and non-action potential dependent synaptic vesicle cycling. iNs were treated with 1 µM TTX and analyzed as in **a** and **b**. *N* = 6 biological replicates. Data are plotted as means ± SD, ****p < 0.0001, ns not significant. Unpaired 2-tailed t-test. **d** Ectopic αS reduces spontaneous synaptic vesicle cycling. Non-evoked synaptic cycling was measured by surface biotinylation in iNs stably expressing αS-wt or vector control. Quantification shows normalized vGlut2 signal expressed as a percentage of the vector control. *N* = 9 biological replicates. Data are plotted as means ± SD, ****p < 0.0001, unpaired 2-tailed t-test. **e** Triplication of endogenous αS in familial PD reduces spontaneous synaptic vesicle cycling. Non-evoked synaptic cycling was measured by surface biotinylation as in **d** in αS triplication or isogenically corrected iNs with quantification of the normalized vGlut2 signal expressed as a percentage of that in the triplication iNs. *N* = 8 biological replicates. Data are plotted as means ± SD, *p < 0.05, unpaired 2-tailed t-test.

Since lower concentrations of KCl allowed for detection of smaller differences in synaptic vesicle cycling (Fig. 1h), we investigated the effects of αS under spontaneous, non-evoked conditions. In these experiments, the biotinylation reagent is incubated with the live cells for 15 min instead of 90 s in the absence of any stimulation, in order to label vGlut2 as it becomes surface exposed with ongoing basal synaptic vesicle cycling. The technique has previously been validated for this purpose^18^. Here, we defined “spontaneous” as both in the absence of exogenous stimulation and without the use of tetrodotoxin (TTX) to block spontaneous action potentials. Thus, the observed signals represent a combination of “miniature” and spontaneous action potential driven events. We performed experiments to confirm that the method measures non-evoked cycling in iNs. First, we treated neurons with botulinum toxin serotype A (BoNT-A), which selectively cleaves the SNARE protein SNAP-25, thus inhibiting vesicle exocytosis. In these cells, non-evoked surface vGlut2 was significantly reduced by BoNT-A treatment, while surface N-cadherin remained unaffected (Fig. 2a). Remaining vGlut2 at the surface with BoNT-A treatment may be explained by the presence of residual SNAP-25 (see the “total” SNAP-25 blot in Fig. 2a), allowing some, albeit reduced, exocytosis to occur. Next, we performed our method both at physiological temperature as before and at 4 °C, where synaptic cycling is significantly reduced, but the reaction of maleimide-PEG2-biotin to surface proteins remains efficient^18^. Surface vGlut2 was accordingly decreased at 4 °C, consistent with reduced cycling, whereas the extent of surface N-cadherin labeling was independent of temperature, as expected (Fig. 2b). To determine the relative contribution of spontaneous miniature events and action potential driven vesicle release, we measured synaptic vesicle cycling in the presence or absence of TTX, which blocks action potentials (Fig. 2c). The approximately 25% reduction in signal with TTX reflected the relative contribution of action potentials to the total signal observed. Taken together, these results support the specificity of the assay in detecting basal, non-evoked synaptic cycling, consistent with prior results in rat neurons^18^.

Having established the applicability of our approach to measuring non-evoked (spontaneous) synaptic vesicle cycling, we asked whether there was an effect of αS in the same iNs we had used for stimulated cycling (Fig. 1e–h). In iNs over-expressing αS, we showed significantly reduced cycling compared to vector control (Fig. 2d). In αS triplication neurons, we also found significantly decreased cycling compared to the isogenic corrected control (Fig. 2e). The degree of change for triplication vs corrected (Fig. 2e) was not as pronounced as that for αS over-expressing vs vector (Fig. 2d), likely due to the relatively modest degree of increased endogenous αS in the triplication compared to the αS-transduced cells.

In conclusion, our work introduces a robust iPSC-based assay platform for measuring synaptic vesicle activity in human neurons that may easily be adopted by laboratories without investments in major equipment. Using this method, we demonstrated that similar to its effects in hippocampal rodent neurons, over-expressed αS restricts synaptic vesicle cycling in human cortical neurons. We then took this a step further by showing for the first time that endogenous triplication of the αS gene in patient-derived cortical neurons inhibits evoked synaptic vesicle cycling. Finally, taking advantage of the unique attributes of our approach, we uncover a role of αS in reducing spontaneous, non-evoked synaptic vesicle cycling in these same cells. Thus, using a technically facile technique, we have gleaned important insights into the effects of disease-associated αS on presynaptic mechanisms in human neurons.

## Methods

### Antibodies

Antibodies used included actin (mouse, Sigma A3853), αS (rabbit, Abcam ab138501, clone MJFR1), beta-3-tubulin (chicken, Abcam ab41489), GAPDH (rabbit, Abcam ab181602), Homer1 (rabbit, synaptic systems 160003), N-cadherin (rabbit, Abcam ab76011), PMCA1 (rabbit, Abcam ab190355), POU3F2/Brn2 (rabbit, CST mab#12137), SNAP-25 (rabbit, Abcam ab5666), vGlut2 (mouse, Abcam ab79157 clone 8G9.2).

### Induced neurons (iNs)

The established BR33^17^ and 2132^30^ iPSC lines from clinically characterized healthy individuals were transduced with TetO-Ngn2-Puro for Neurogenin-2 mediated differentiation as before^16,26^. αS-wt over-expressing iPSCs generated by lentiviral transduction in the 2132 cell background have been described^28^. The *SNCA* triplication and corrected iPSC lines (AST23) were obtained through EBISC thanks to a generous gift from the Kunath lab (University of Edinburgh)^31^. Tissue culture surfaces were coated with an initial layer of poly-L-ornithine (Sigma P3655) at 20 µg/mL and laminin (Gibco 23017015) at 5 µg/mL in PBS overnight in the incubator at 37 °C. On the day of plating, the poly-L-ornithine and laminin in PBS were removed by aspiration and a second layer of Matrigel Basement Membrane Matrix (Corning 354234) was applied at a concentration of 0.2 mg/mL in DMEM/F12 (a total of 0.5 mL per well of a 24-w plate was used). Media for plating iNs consisted of a base NBM, composed of Neurobasal (Gibco 21103049) supplemented with 20% dextrose, 1× Glutamax (Gibco 35050061), and 1× MEM Non-Essential Amino Acids Solution (Gibco 11140035), plus 1× B27 supplement (Gibco 17504044), 2 µg/mL doxycycline (Sigma D9891), 5 µg/mL puromycin (Gibco A1113803), 10 ng/mL BDNF (Peprotech 450-02), 10 ng/mL CNTF (Peprotech 450-13), 10 ng/mL GDNF (Peprotech 450-10), 10 µM ROCK inhibitor (Stemcell Technologies 72304), and 1 µg/mL laminin (Gibco 23017015). iNs were maintained in the same media, except that no ROCK inhibitor or laminin was added. Half media changes were performed thrice weekly. iNs were used for experiments at between DIV21-24.

### Surface biotinylation synaptic vesicle cycling assay

The method is based on that of Xu and colleagues^18^ with changes as follows. iNs between DIV21-24 in 24-well plates (plated at a density of 180,000 cells per well) were pre-equilibrated in the incubator for 45–60 min in extracellular solution (ECS) containing (in mM): 140 NaCl, 2.7 KCl, 1.7 CaCl_2_, 1.0 MgCl_2_, 10 HEPES, and 33 glucose, pH 7.4. ECS was removed by aspiration and replaced with ECS containing either 1 mg/mL EZ-link Maleimide-PEG2-Biotin (Thermo 21901BID) or 1 mg/mL EZ-link Sulfo-NHS-SS-Biotin (Thermo 21331) with varying amounts of KCl as follows. For 90 mM KCl stimulation conditions, the ECS composition was: 31.5 NaCl, 90 KCl, 1.7 CaCl_2_, 1.0 MgCl_2_, 10 HEPES, and 33 glucose, pH 7.4. For 30 mM KCl stimulation conditions, the ECS composition was: 91.5 NaCl, 30 KCl, 1.7 CaCl_2_, 1.0 MgCl_2_, 10 HEPES, and 33 glucose, pH 7.4. iNs were bathed in these solutions for either 60 (Fig. 1b, c, Supplementary Figs. 2 and 3) or 90 s (all other experiments; times are also indicated in the figure legends) in the incubator to biotinylate exposed vGlut2 during KCl-induced depolarization. For spontaneous synaptic vesicle cycling experiments, iNs were pre-equilibrated in ECS as above and then surface biotinylated in ECS containing 1 mg/mL maleimide-PEG2-biotin for 15 min. Sulfo-NHS-SS-biotin or maleimide-PEG2-biotin containing solutions were then removed by aspiration and cells washed twice in ice-cold ECS to remove unreacted biotinylation reagent. Plates were kept on ice in ECS for an additional 30 minutes after washing. iNs were then lysed in 120 µL of a modified RIPA lysis buffer containing 50 mM Tris pH 7.4, 1% Triton X-100, 0.1% sodium deoxycholate, 0.1% SDS, 150 mM NaCl, 1 mM EDTA, supplemented with protease inhibitors (Halt protease inhibitor cocktail, Thermo 78429). After saving 10% of the lysate for input, the remainder was added to 40 µL of washed neutravidin-agarose resin (Thermo/Pierce 29200) with volume topped up to 450 µL with mRIPA for an overnight pulldown with end-over-end rotation at 4 °C. After pulldown, neutravidin-agarose beads were washed 3 times in mRIPA and aspirated to dryness. Biotinylated proteins were eluted with the addition of 2× LDS sample buffer containing 2.5% beta-mercaptoethanol plus heating to 95 °C for 5 min. Samples and inputs were then resolved by SDS-PAGE.

### Immunoblotting

Samples were loaded and run on NuPAGE 4–12% Bis-Tris gels (Thermo/Invitrogen). Transfers onto nitrocellulose membranes were performed using template P0 on an iBlot 2 (Thermo/Invitrogen). For experiments involving detection of αS, membranes were then fixed for 15 min in 4% PFA (in PBS). Blocking was done using 5% milk in PBS. Membranes were incubated in primary antibody in 5% milk in PBST overnight at 4 °C. Imaging of membranes was performed using appropriate secondary antibodies followed by scanning on an Odyssey CLx scanner (LI-COR). All blots were processed in parallel and derive from the same experiments.

#### Immunofluorescence microscopy

Coverslips (Electron Microscopy Sciences #7229102) were coated with poly-L-ornithine, laminin, and Matrigel as descrbied above for routine iN cultures. On DIV21, iNs were washed once with PBS, then fixed with 4% paraformaldehyde in PBS for 15 min at room temperature. Following fixation, iNs were washed with PBS for 15 minutes at room temperature then incubated in blocking buffer (2% donkey serum with 0.1% Triton X-100) for 1 h shaking at room temperature. iNs were incubated in primary antibodies diluted in blocking buffer overnight at 4 °C. Following incubation iNs were washed with PBS 3 times for 10–15 min and incubated in secondary antibodies for 1 h at room temperature. Secondaries included Cy2 Donkey anti-rabbit, Cy2 Donkey anti-mouse, Cy3-Donkey anti-mouse, Cy3-Donkey anti-rabbit, Cy5-Donkey anti-chicken 1:2000, (Jackson Immunoresearch). iNs were then washed with PBS 3 times. DAPI (1:1000) staining was performed during the second wash with PBS. Coverslips were mounted and imaged using Zeiss LSM710 confocal microscopy, 40× oil objective and acquired using Zen black software.

### SynaptopHluorin (sypHy) assay

We followed the method as previously described^32^. Briefly, pre-treated glass coverslips #1.5, 12 mM (Electron Microscopy Sciences 7229102) were placed in 24-well plates and coated with poly L-ornithine, laminin, and Matrigel as described above in the “iN” section. iNs were plated at a density of 180,000 cells per well and maintained also as described. On DIV 18, cells were transduced at a multiplicity of infection of with lentivirus encoding FSW-sypHy, a gift from Pascal Kaeser. Lentiviral particles were generated by Alstem (Richmond, CA). On DIV21, coverslips were transferred to an open diamond bath imaging chamber (Warner R-25, 64-0232) with a Zeiss stage adapter (Warner SA-20KZ-AL, 64-2415). Cells were perfused with the same pre-equilibration and high 90 mM KCl stimulation solutions we used for the surface biotinylation assay described above. sypHy fluorescence was measured over the course of a 140 frame time series in the Zen Black software with the 40× oil objective. Cells were perfused with ECS from 0 to 30 s, stimulated with 90 mM KCl from 30 to 90 s, and unquenched with ammonium chloride solution containing (in mM): 90 NaCl, 50 NH_4_Cl, 2.7 KCl, 1.7 CaCl_2_, 1 MgCl_2_, 10 HEPES, 33 glucose, pH 7.4.

For data analysis, the FIJI time series analyzer was used to quantify fluorescence intensity in regions of interest (ROIs) over time. ROIs were areas containing synaptic puncta. For each ROI, the following intensities were measured: F_0_ (mean fluorescence at baseline); F_stim_ (mean fluorescence with KCl stimulation); and F_NH4Cl_ (max fluorescence at unquenching). Then ∆F = F_stim_ − F_0_ was calculated. We only considered those puncta with a fold change of F_NH4Cl_ to F_0_ greater than 2 and with ∆F > F0 for subsequent analyses. ∆F was then calculated as a percentage of F_NH4Cl_ to determine the response to KCl stimulation.

### Dyngo treatment

Dyngo-4a (Abcam ab120689) was added to cells at a final concentration of 30 µM during the pre-equilibration step of the synaptic cycling assay in ECS. Subsequent biotinylation also occurred in the presence of 30 µM Dyngo-4a in either 2.7 or 90 mM KCl containing ECS.

### Botulinum toxin treatment

Botulinum toxin serotype A (BoNT-A) complex (Metabiologics/Object Pharma #100) was reconstituted in PBS and added to cells at a concentration of 20 pM overnight.

### Tetrodotoxin (TTX) treatment

Tetrodotoxin citrate was purchased from Cayman Chemical (cat #14964), reconstituted in water, and added to cells at a concentration of 1 µM in ECS during the 45–60 min pre-equilibration step of the synaptic cycling assay. Biotinylation solutions containing maleimide-PEG2-biotin also contained 1 µM TTX during the 15 min non-stimulated labeling step.

### Reporting summary

Further information on research design is available in the Nature Research Reporting Summary linked to this article.

### Supplementary information

Supplementary Figures

Reporting summary

## Data Availability

All data generated (and non-commercially available tools used in experiments) are included in this published article and its Supplementary Information section.

## Code availability

Zen Black software was used to acquire immunofluorescence images and video for the sypHy assay. LiCor ImageStudio was used to acquire near infra-red fluorescent Western blot images. Image analysis of immunofluorescent data was done using FIJI; analysis of Western blot images was done with LiCor ImageStudio. Statistical analyses were performed using Graphpad Prism 10.
