# Supplementary material for: A stem cell-based assay platform demonstrates alpha-synuclein dependent synaptic dysfunction in patient-derived cortical neurons
Source: NPJ Parkinsons Dis. 2024 May 21;10:107. doi: 10.1038/s41531-024-00725-y (PMC11109103; doi:10.1038/s41531-024-00725-y)
Supplement: Supplementary file 1 — Supplementary Figures [file 41531_2024_725_MOESM1_ESM.pdf]

Supplementary Figure 1

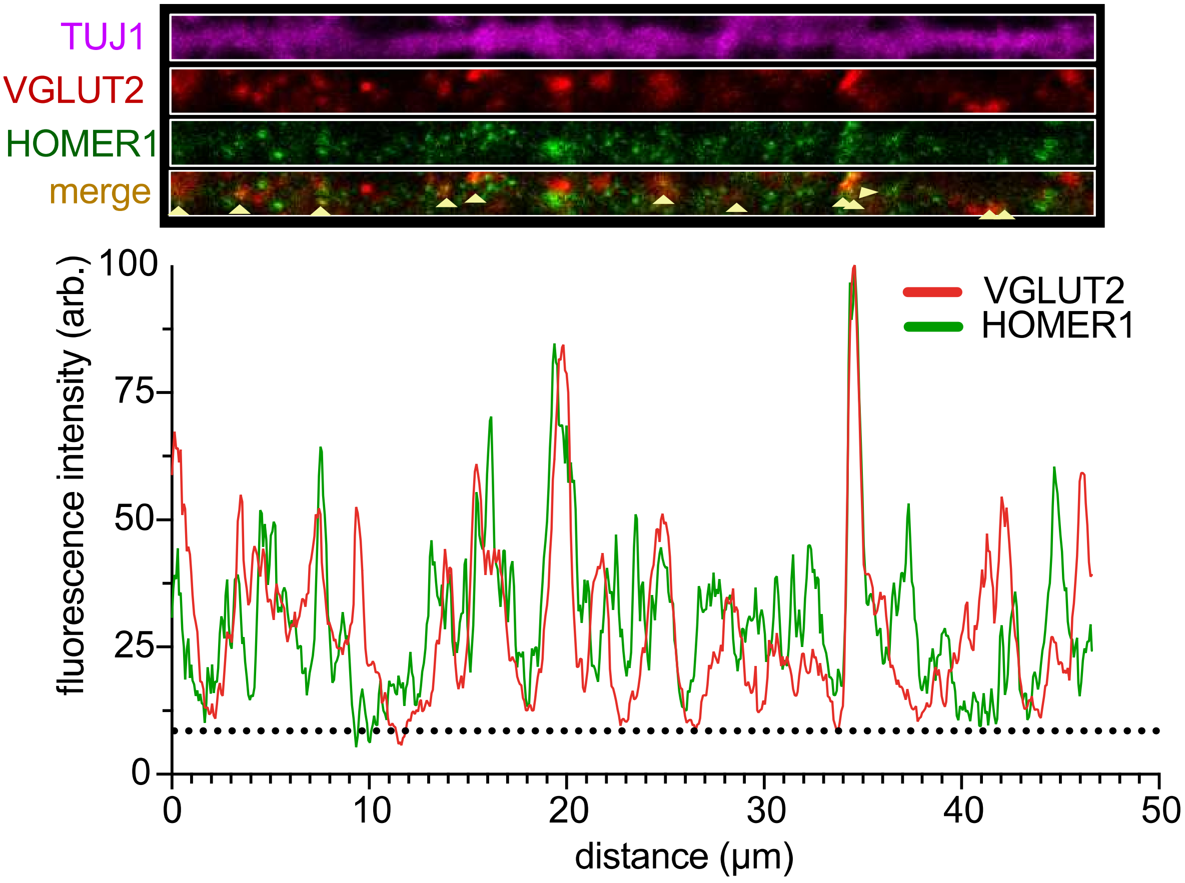

Supplementary Figure 1. Induced neurons (iNs) form synapses. *Top,* immunofluorescence for presynaptic vGlut2 (red) and postsynaptic marker Homer1 (green) in BR33 iNs (DIV21). Images are from a single plane within an iN process (beta-3-tubulin/TUJ1, magenta)**.** *Bottom,* line scan showing relative fluorescence intensity of HOMER1 (green) and VGlut2 (red) for the same 46μm region. Arrowheads indicate areas of overlapping peak red and green puncta intensity consistent with synaptic structures. Dotted line represents background fluorescence.

Supplementary Figure 2

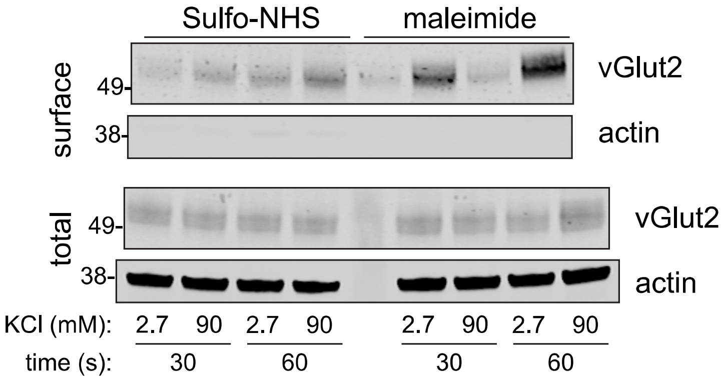

Supplementary Fig. 2 Comparison of membrane impermeable surface biotinylation reagents. iNs were stimulated for the indicated times with 90 mM KCl-containing ECS and surface biotinylation was performed using either sulfo-NHS-SS-biotin or maleimide-PEG2-biotin followed by pulldown, elution, and WB for the indicated proteins.

Supplementary Figure 3

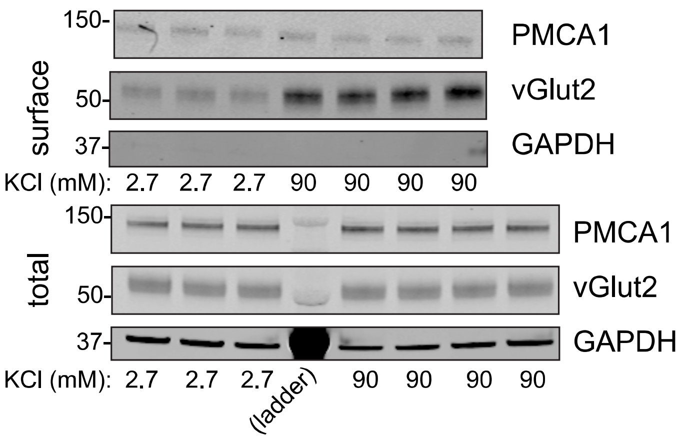

Supplementary Fig. 3 Biotinylation of constitutive surface proteins is unaffected by KCl-mediated depolarization. iNs were stimulated with 90 mM KCl for 60 s and subjected to surface biotinylation with maleimide-PEG2-biotin followed by pulldown, elution, and WB for PMCA1 and the indicated proteins.

Supplementary Figure 4

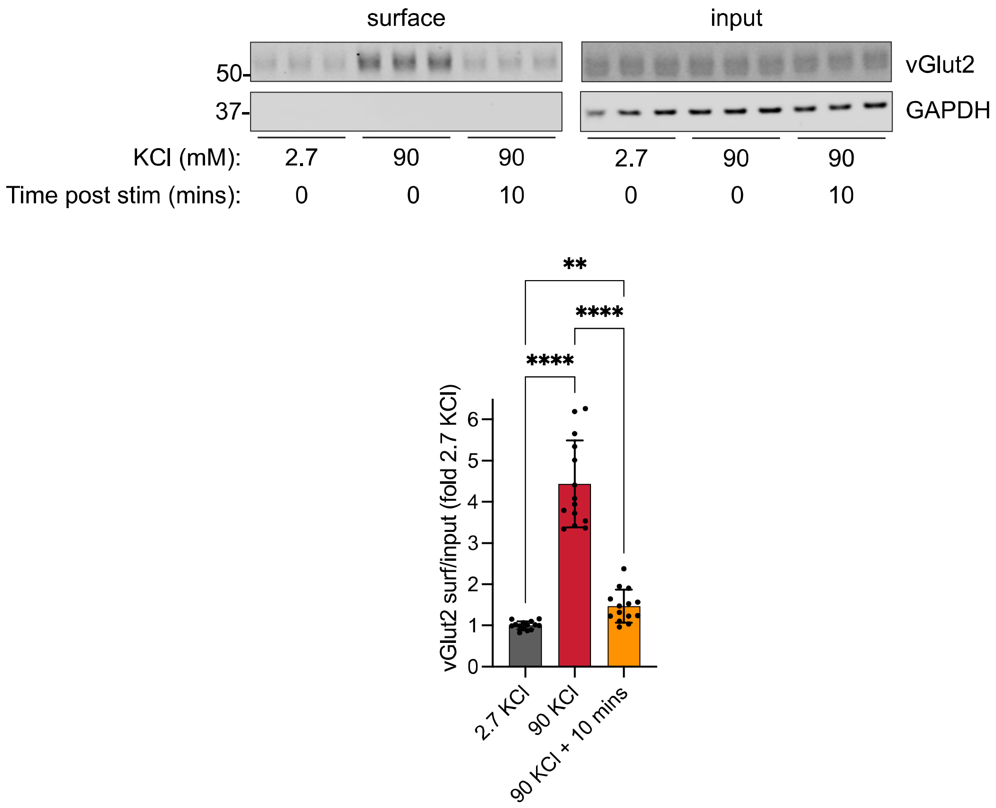

Supplementary Fig. 4 Surface biotinylation of vGlut2 is reduced following removal of 90 mM KCl stimulation. iNs were left in basal 2.7 mM KCl or stimulated with 90 mM KCl for 90 s and surface biotinylated with maleimide-PEG2-biotin in the same solution (first 6 lanes). This was deemed time 0. For the last 3 lanes, 90 mM KCl stimulation was carried out in the absence of any maleimide-PEG2-biotin. Solution was removed and cells returned to normal 2.7 mM KCl containing ECS at physiological temperature for 10 minutes. 10 minutes post-stimulation, iNs were subjected to surface biotinylation with maleimide-PEG2-biotin followed by pulldown, elution, and WB for the indicated proteins. N=14 biological replicates. Data are plotted as means -/+ SD, **$p<0.01$, ****$p<0.0001$, 1-way ANOVA with Dunnett's multiple comparisons test.

Unprocessed, uncropped WB for Figure 1

1b

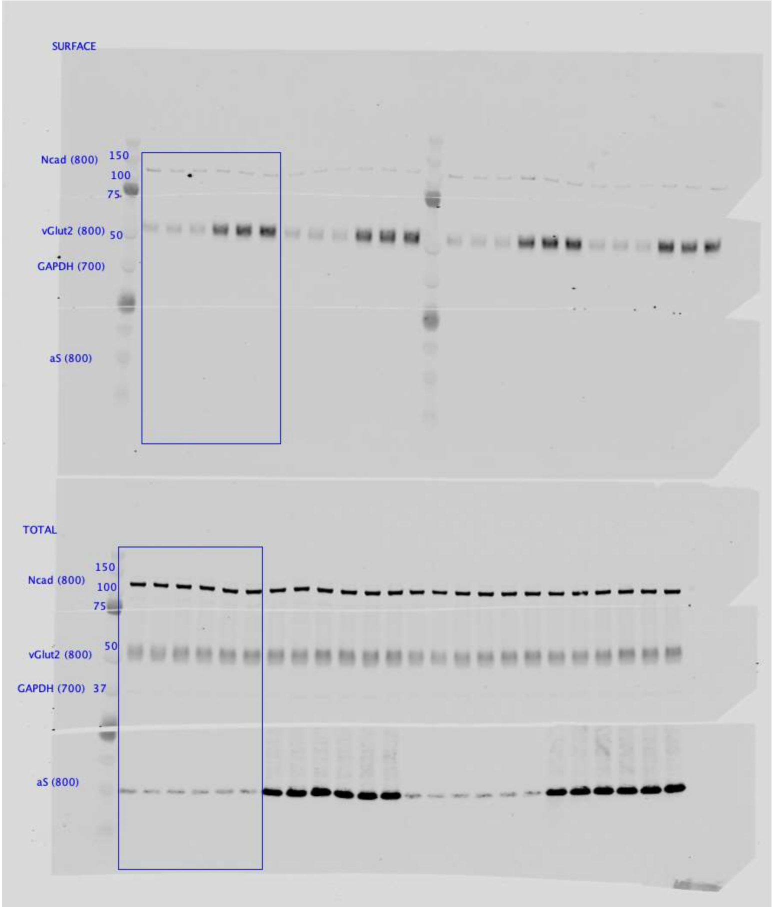

800 channel

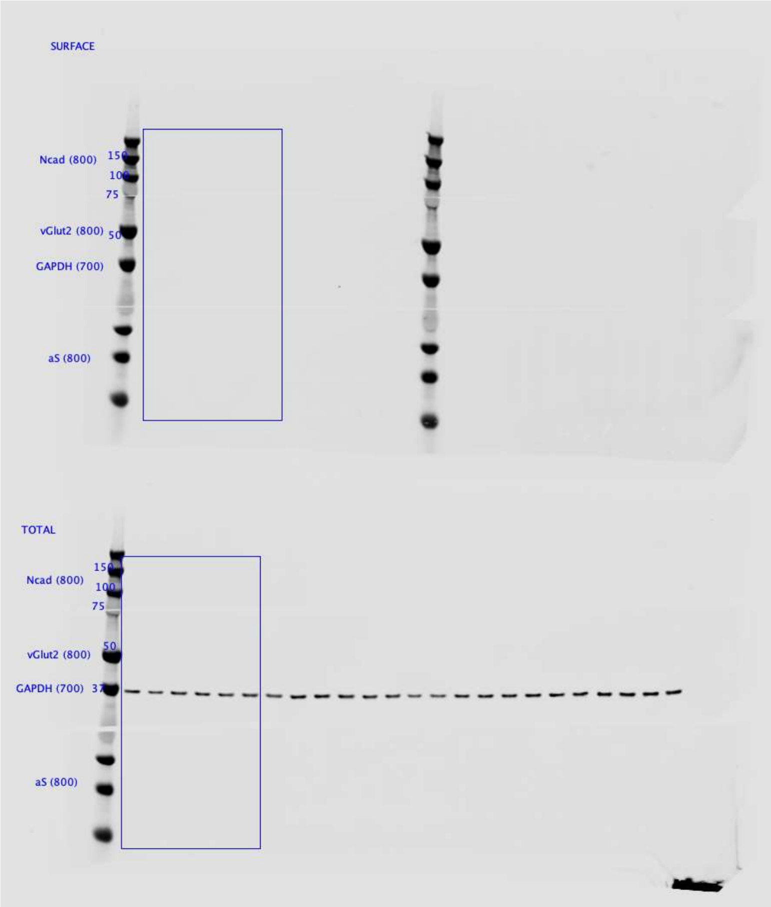

700 channel

1c

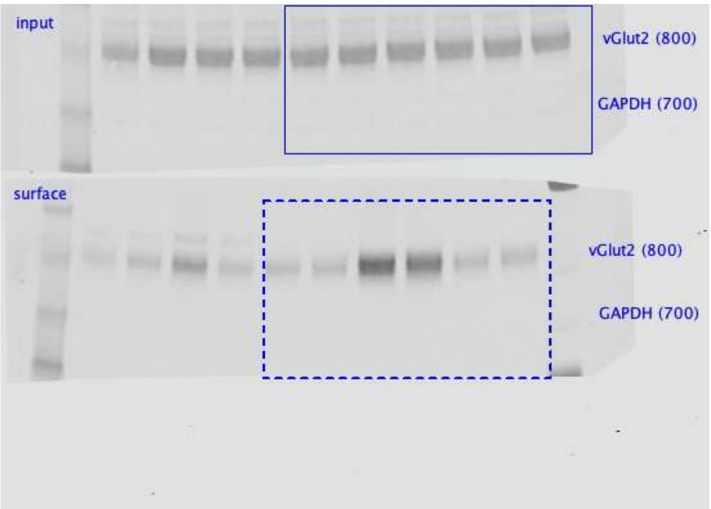

800 channel

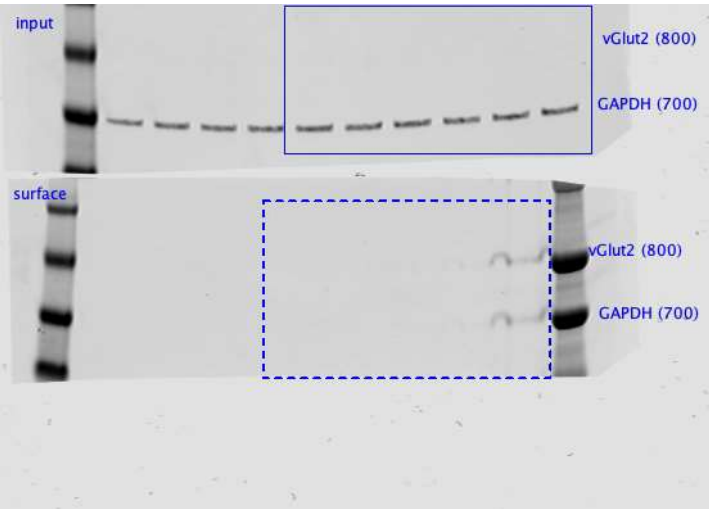

700 channel

Unprocessed, uncropped WB for Figure 1 (cont)

1d

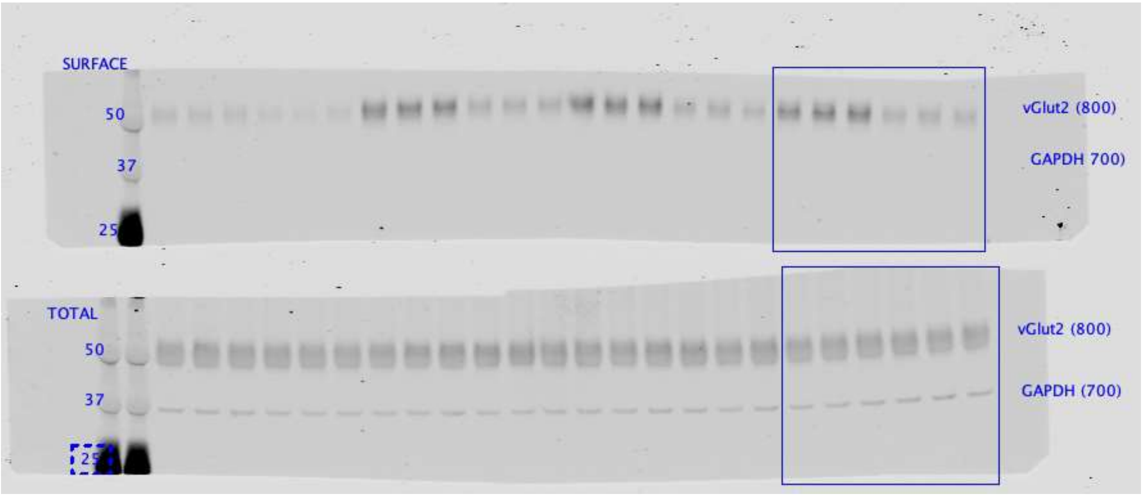

800 channel

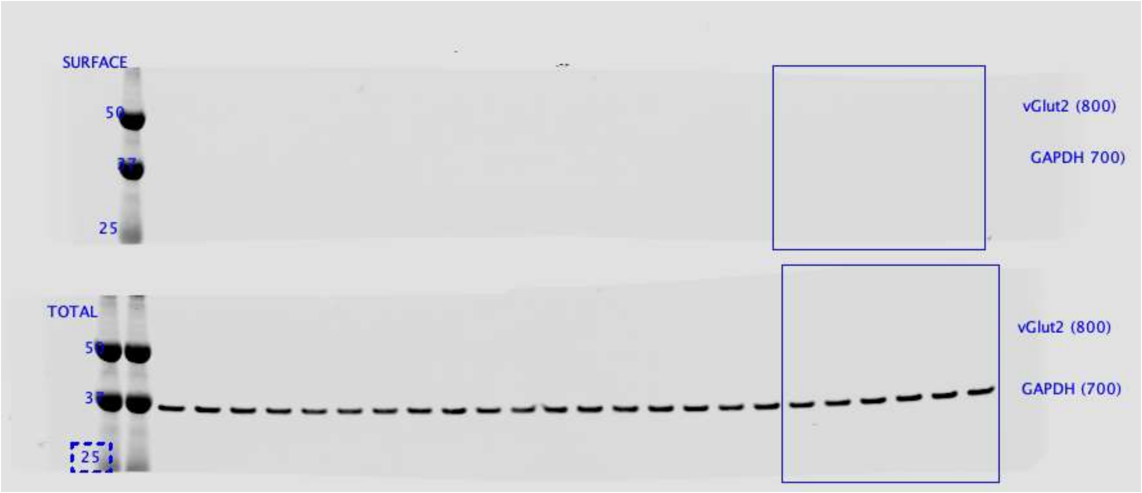

700 channel

Unprocessed, uncropped WB for Figure 1 (cont)

1e

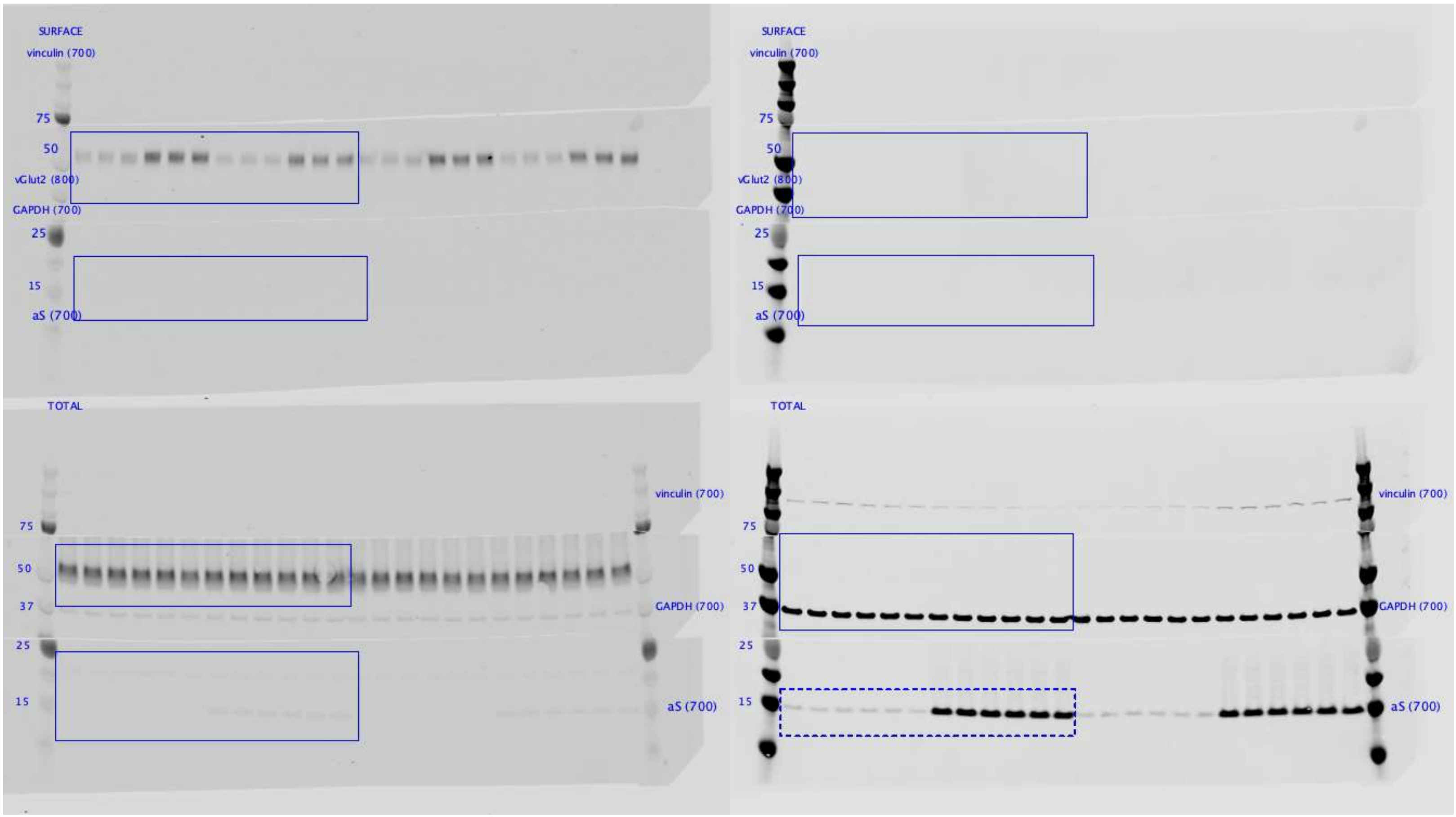

800 channel

700 channel

Unprocessed, uncropped WB for Figure 1 (cont)

1g

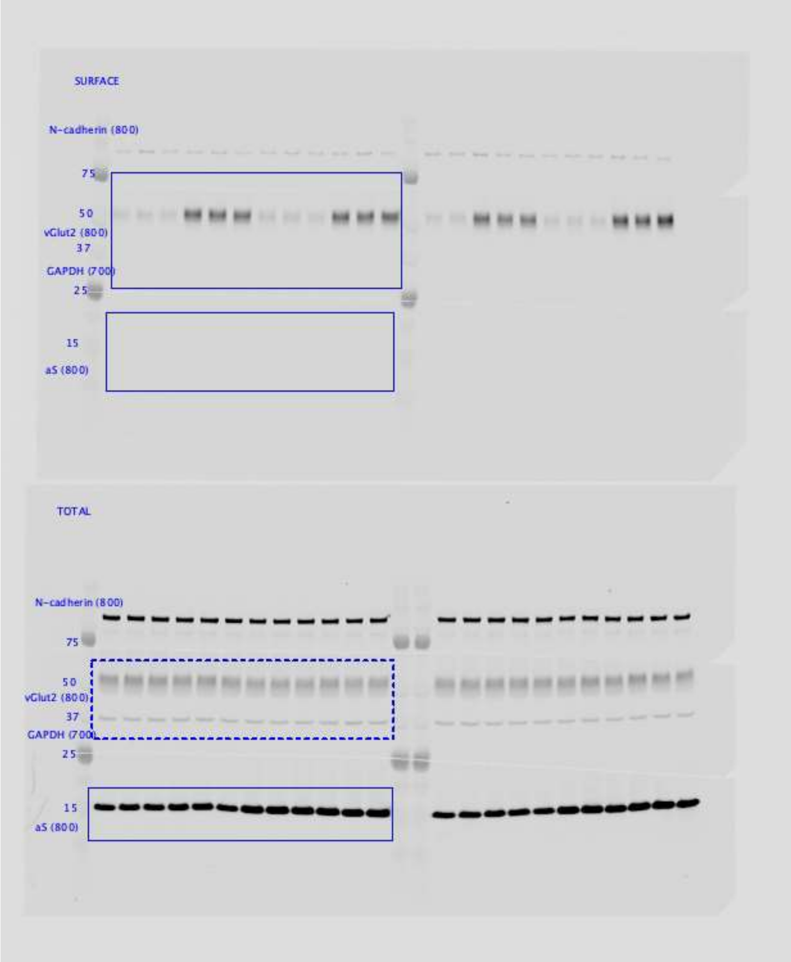

800 channel

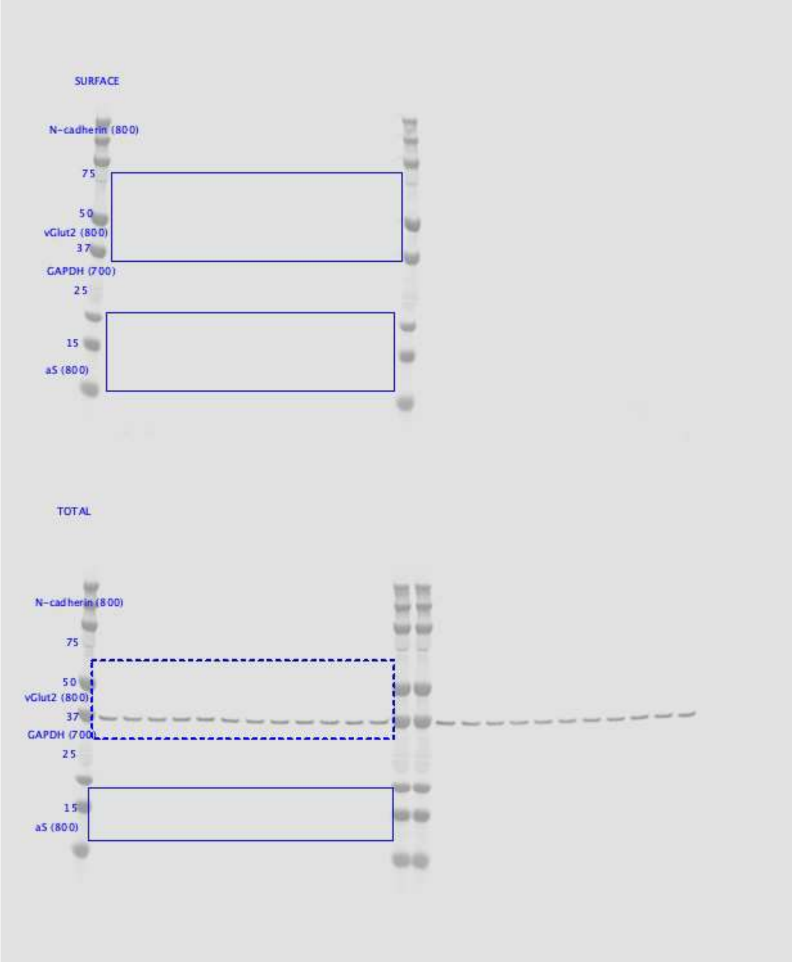

700 channel

Unprocessed, uncropped WB for Figure 1 (cont)

1h

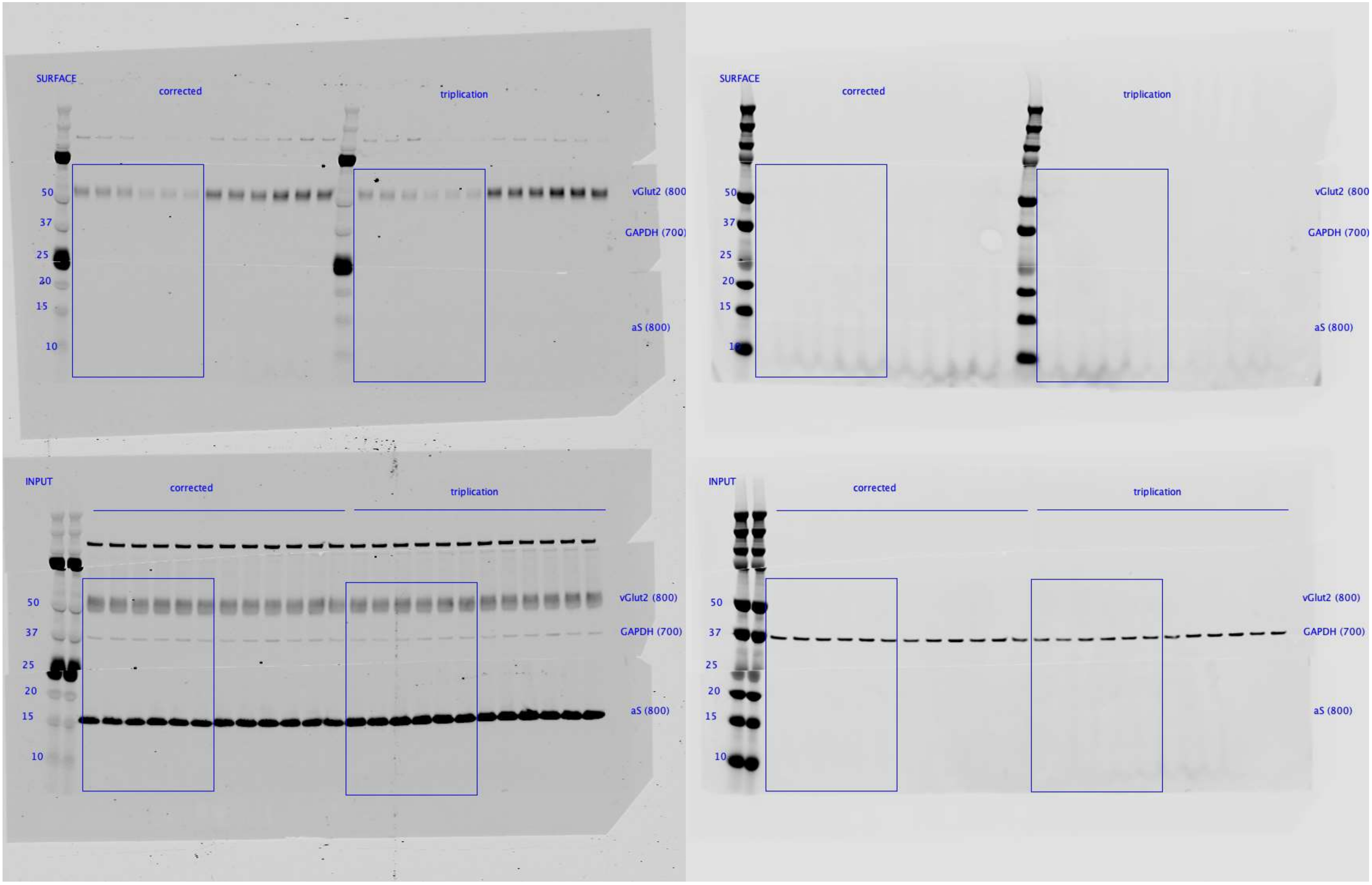

800 channel

700 channel

*Note that for Fig 1h these crops were flipped horizontally to more logically and consistently display 2.7 followed by 30 mM KCl conditions.

Unprocessed, uncropped WB for Figure 2

2a

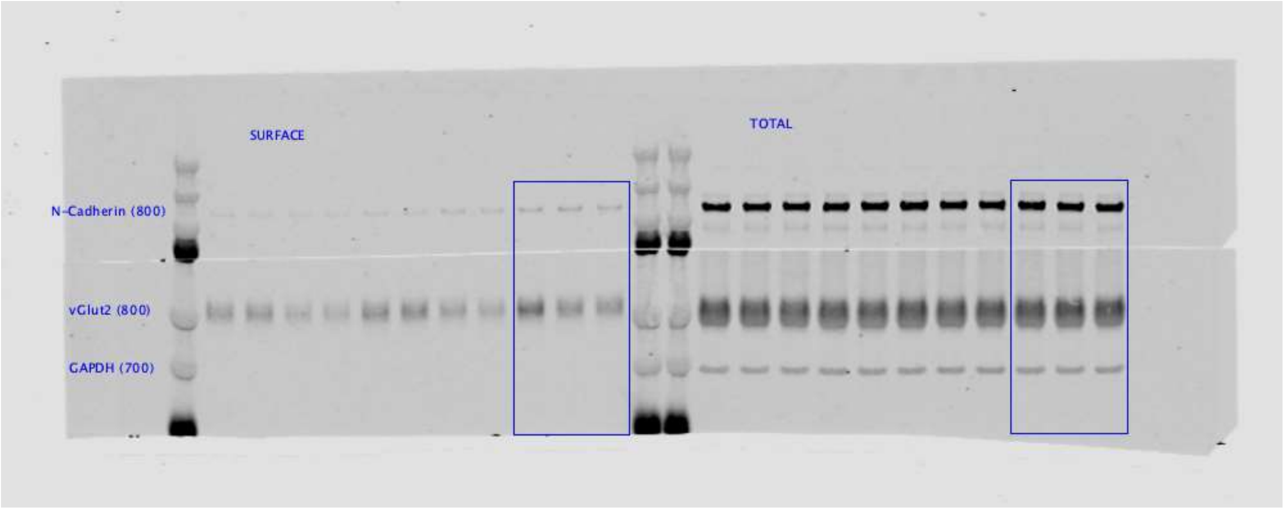

800 channel

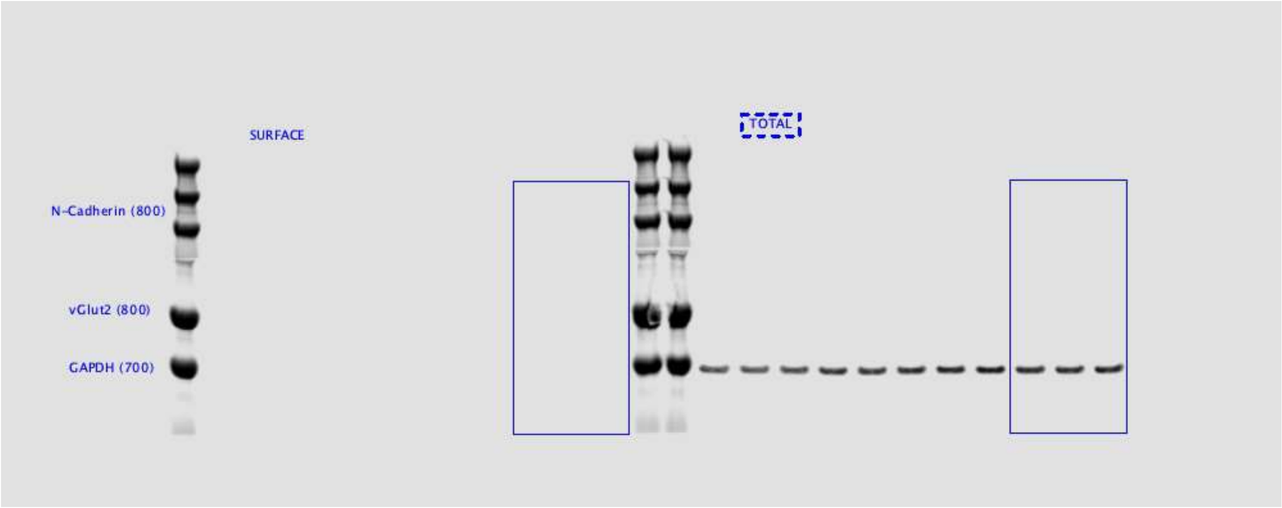

700 channel

Unprocessed, uncropped WB for Figure 2 (cont)

2a (reprobed for SNAP-25)

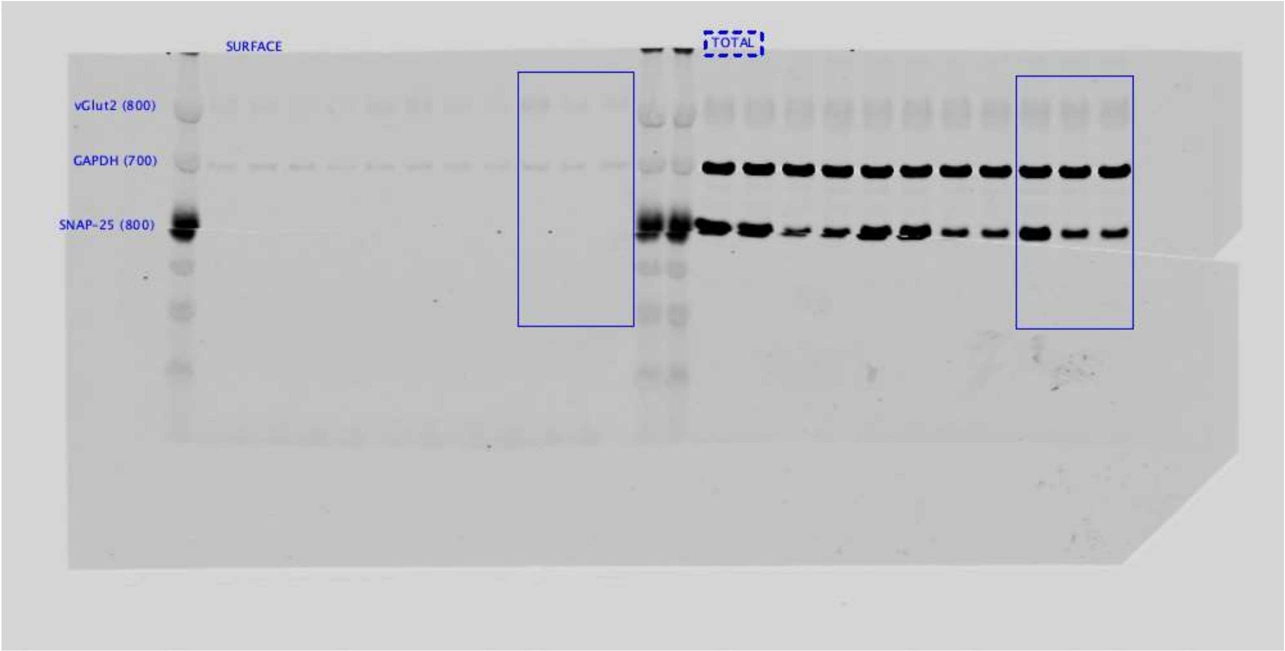

800 channel

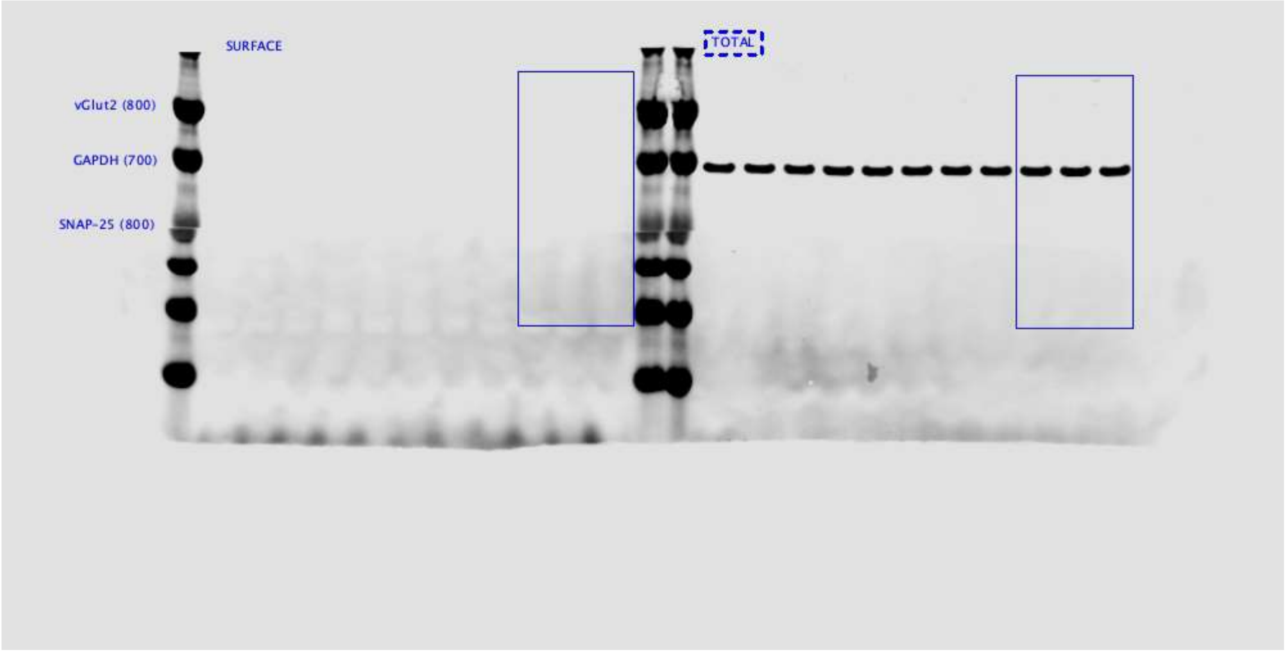

700 channel

Unprocessed, uncropped WB for Figure 2 (cont)

2b

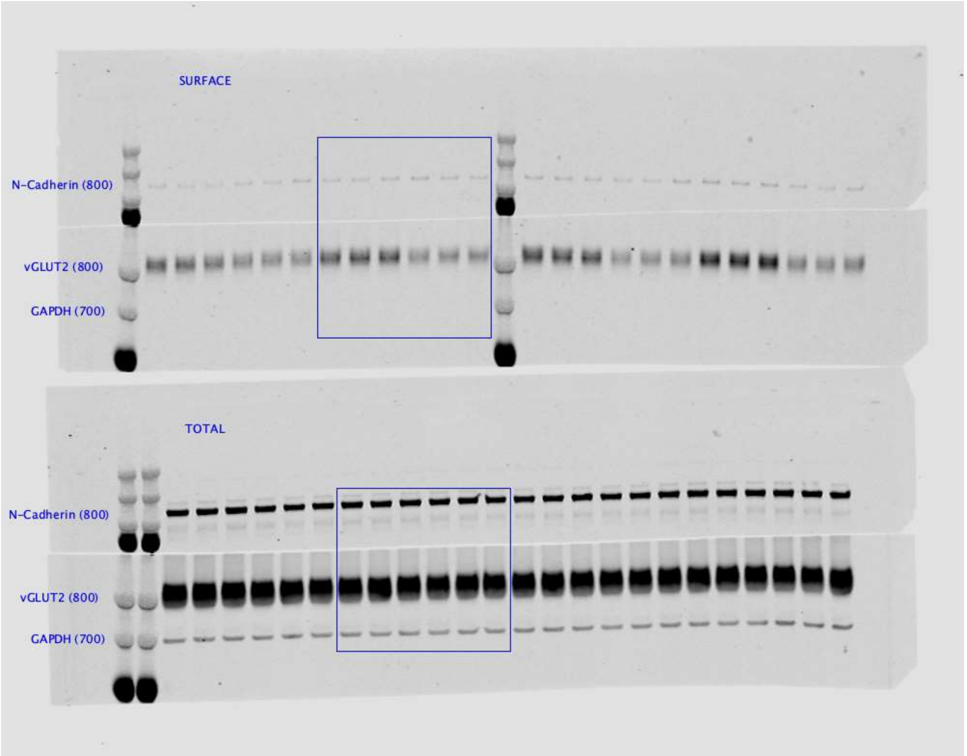

800 channel

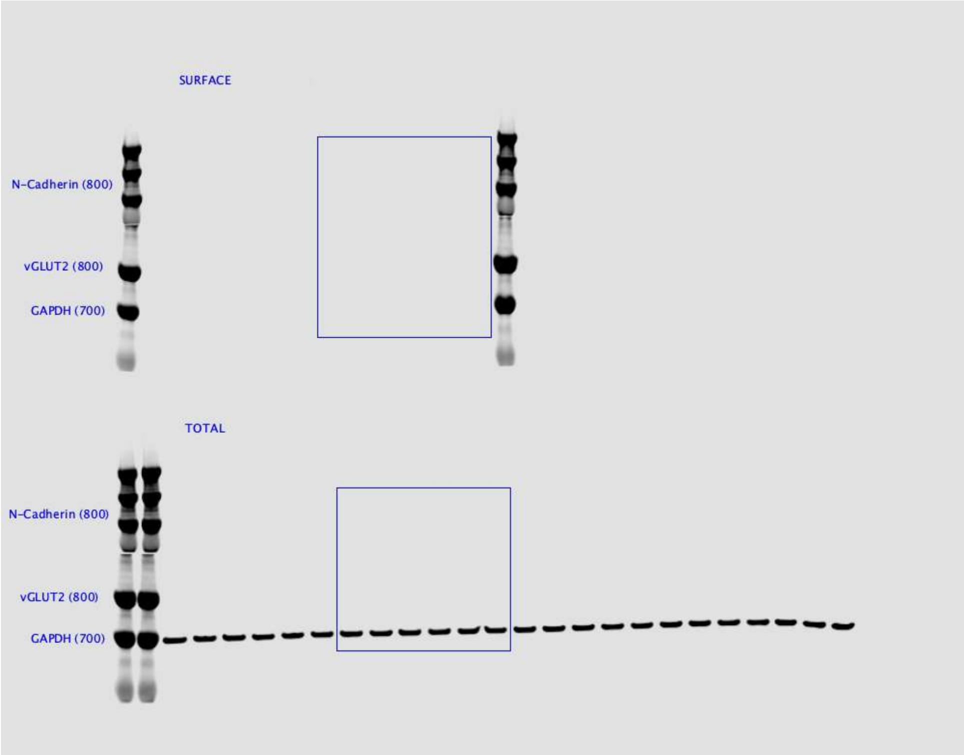

700 channel

Unprocessed, uncropped WB for Figure 2 (cont)

2c

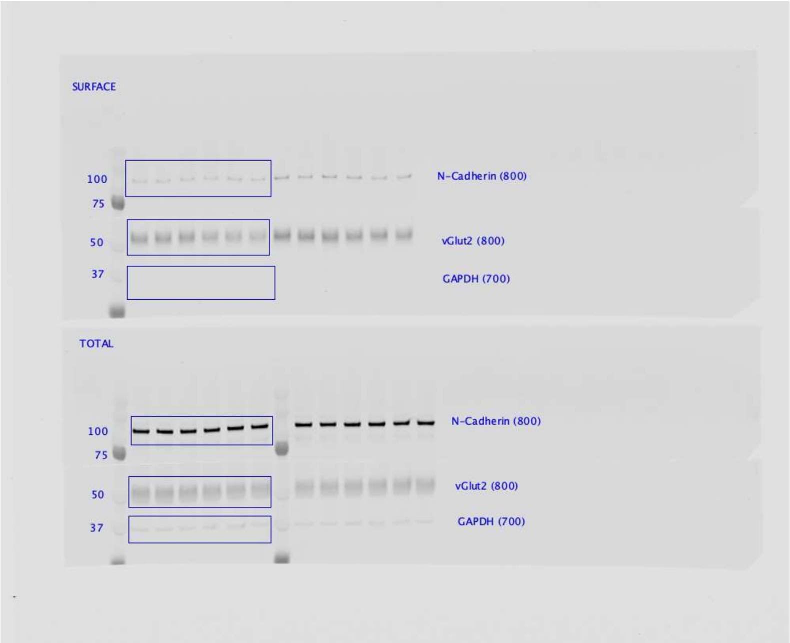

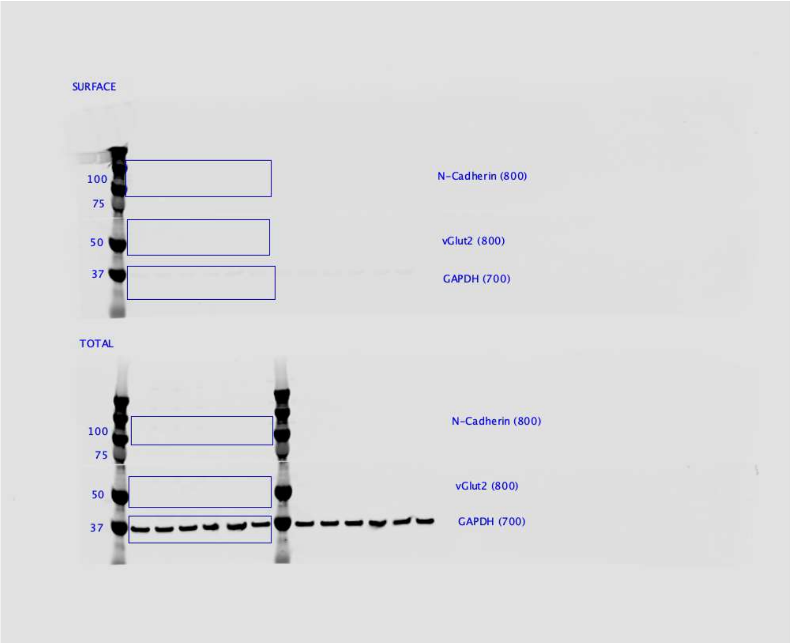

800 channel

700 channel

2d

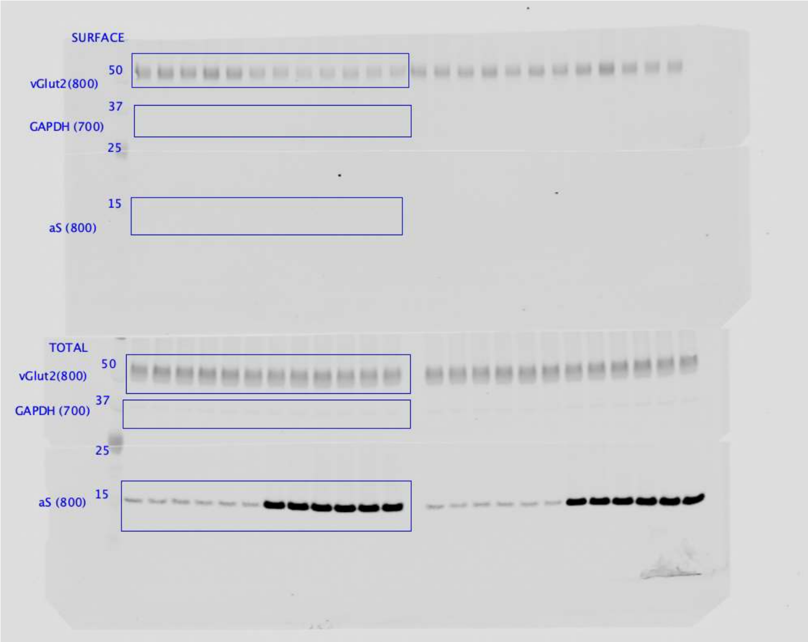

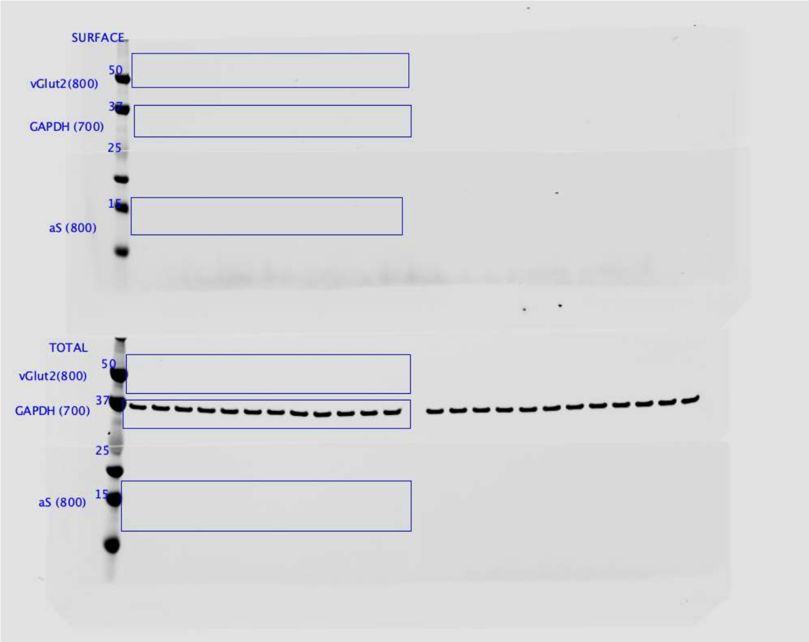

800 channel

700 channel

Unprocessed, uncropped WB for Figure 2 (cont)

2e

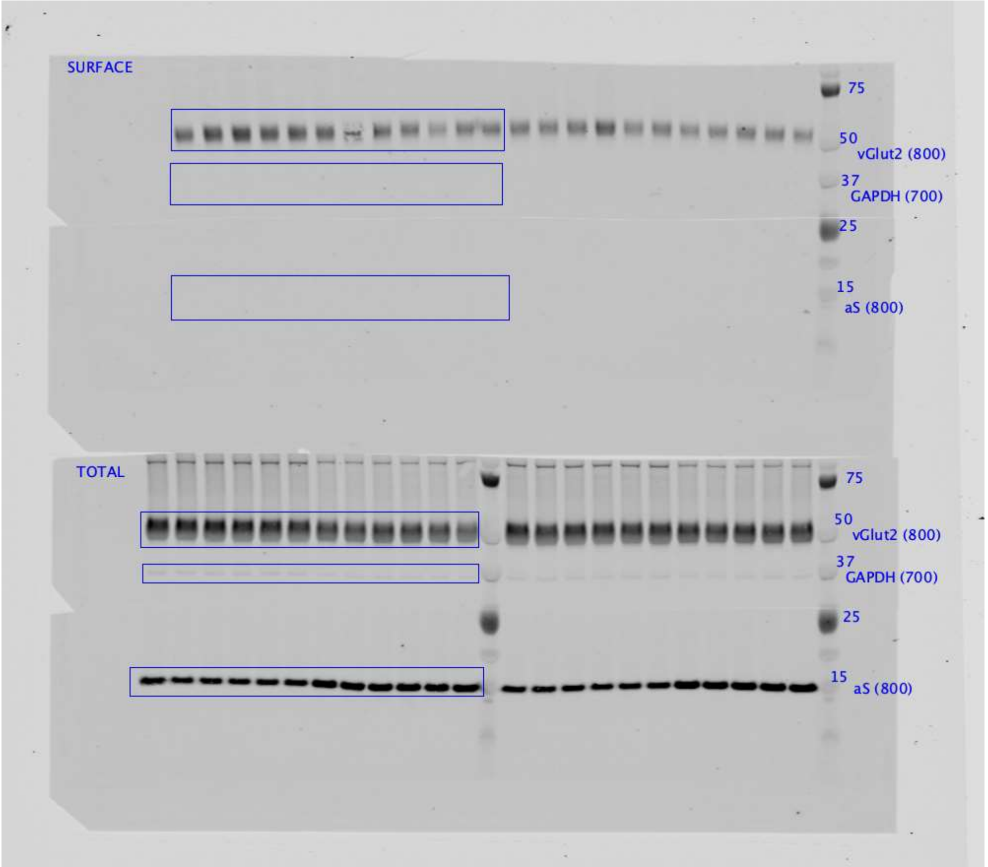

800 channel

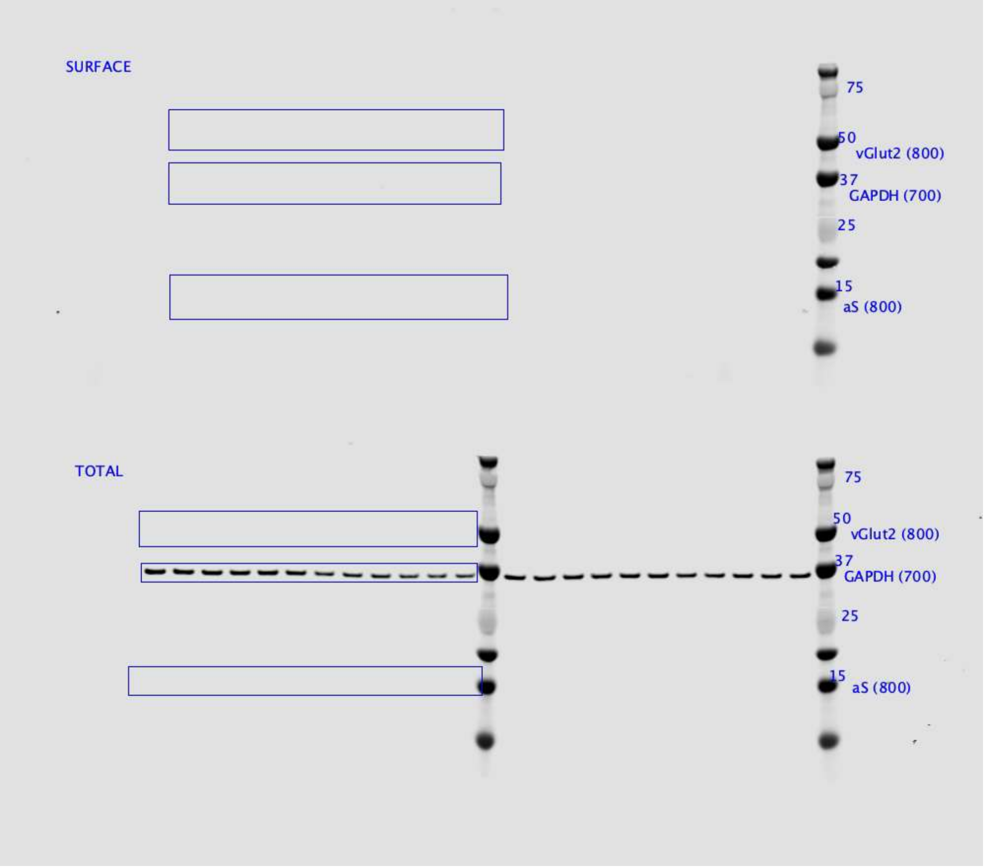

700 channel

Unprocessed, uncropped WB for Supp Figure 2

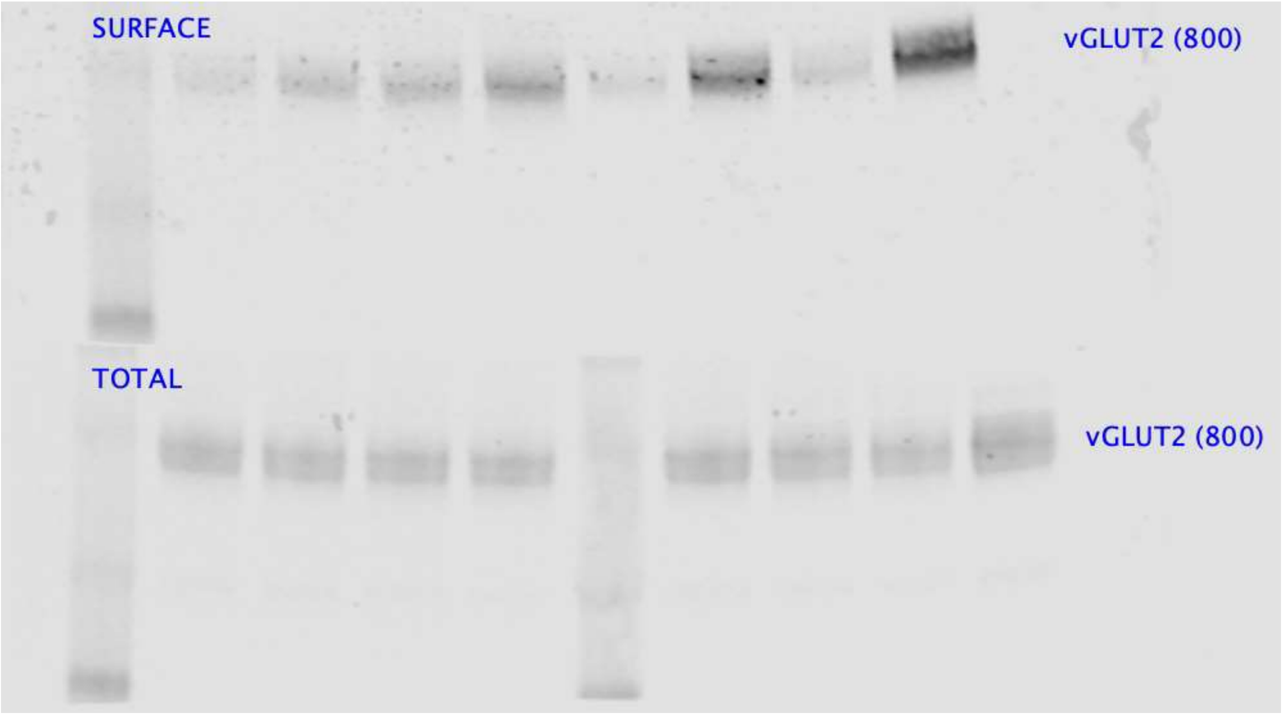

800 channel

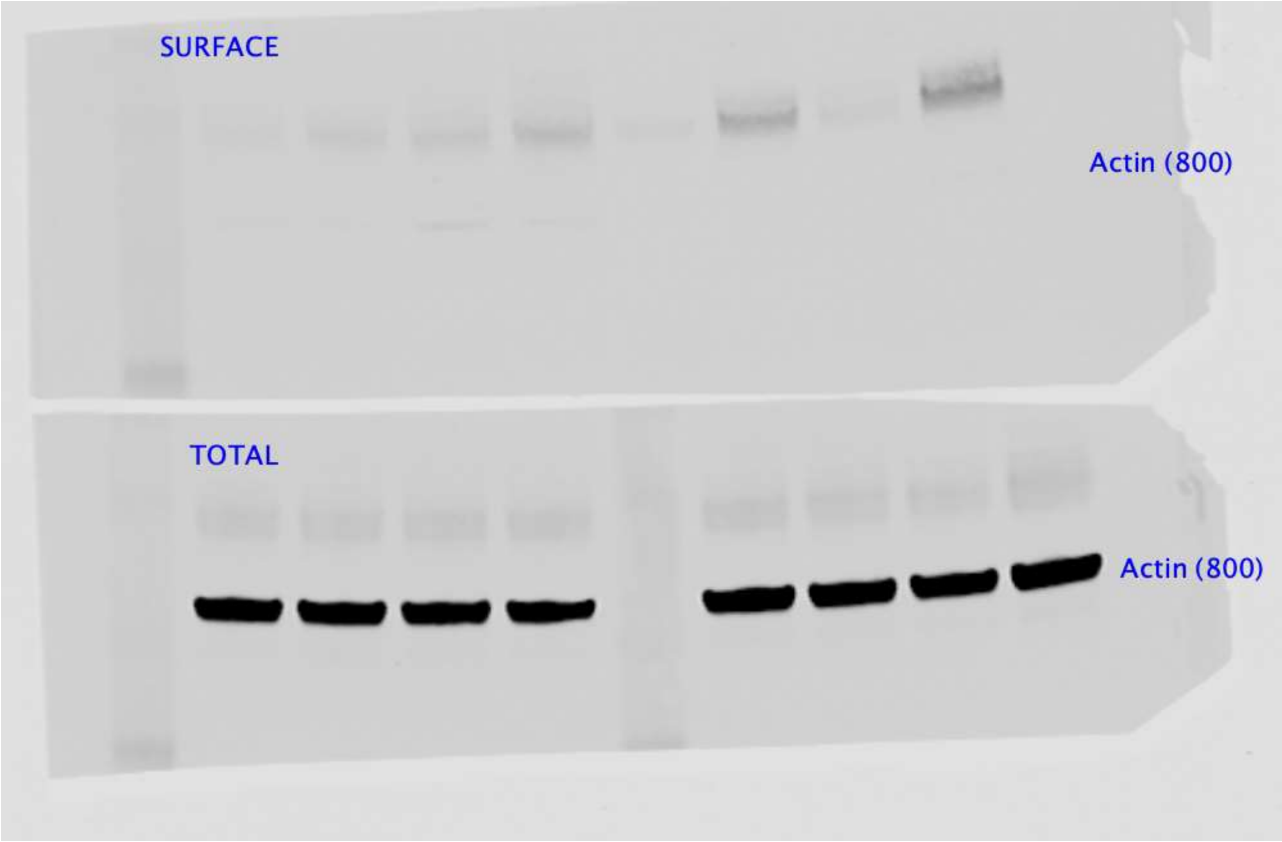

800 channel (reprobed for actin)

Unprocessed, uncropped WB for Supp Figure 3

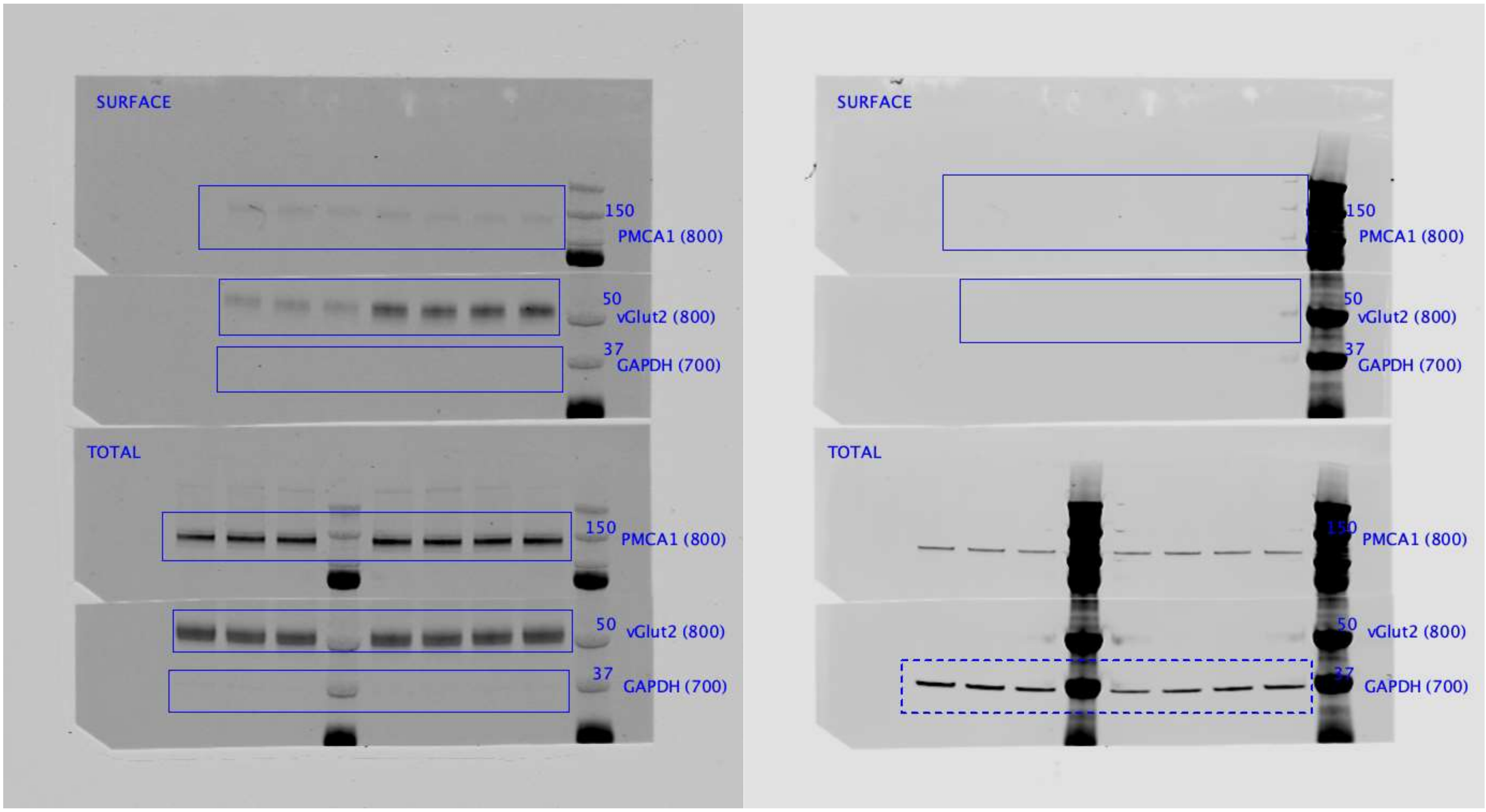

800 channel

700 channel

Unprocessed, uncropped WB for Supp Figure 4

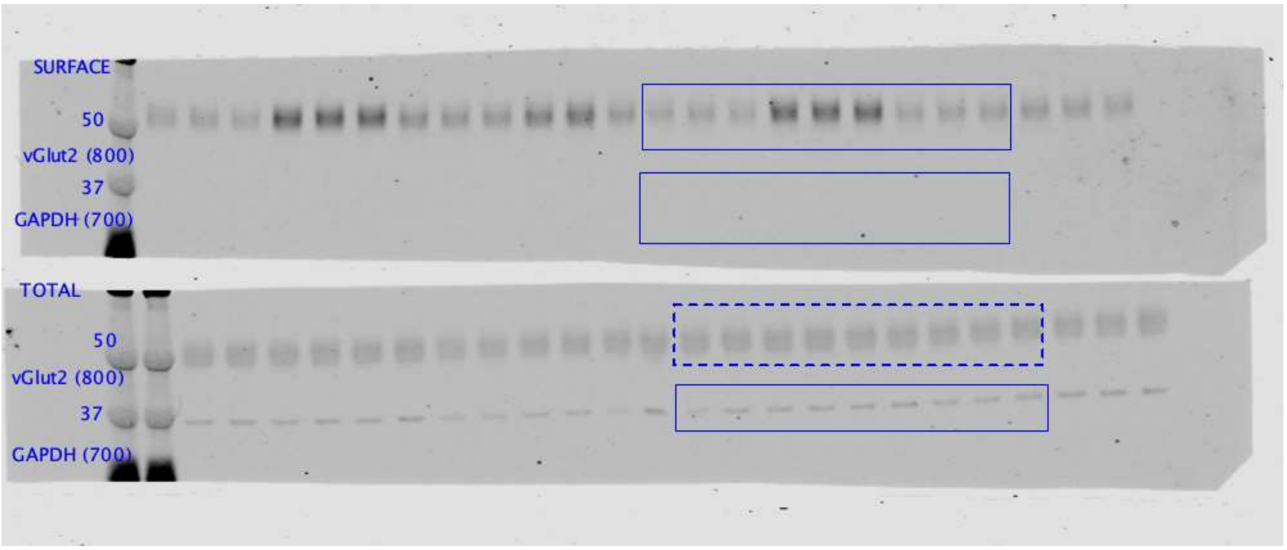

800 channel

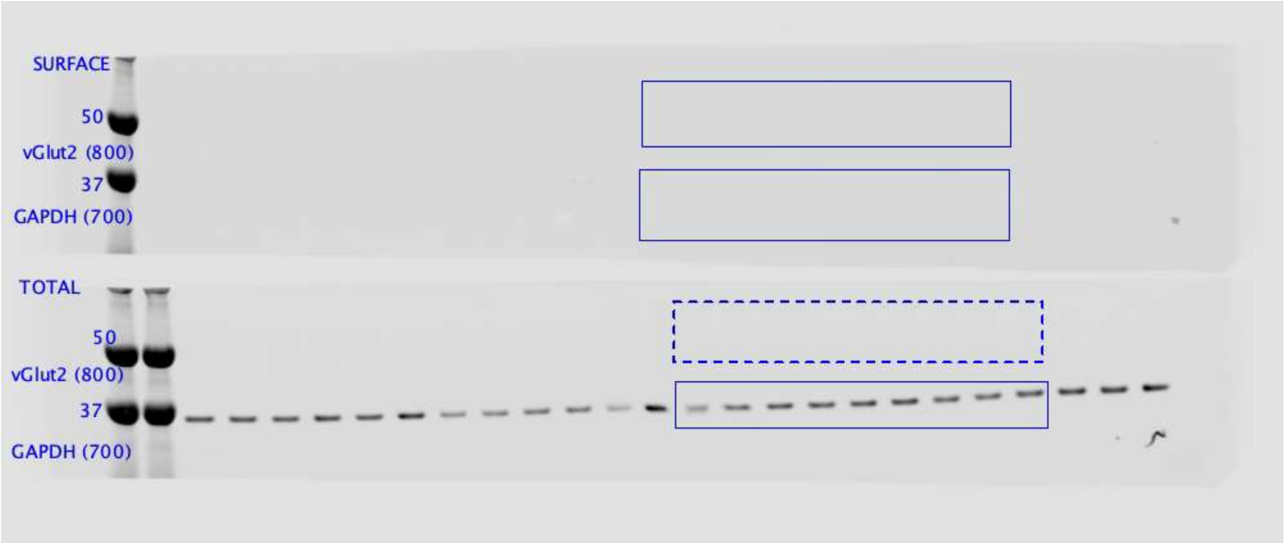

700 channel
