# Supplementary material for: A stem cell-based assay platform demonstrates alpha-synuclein dependent synaptic dysfunction in patient-derived cortical neurons
Source: NPJ Parkinsons Dis. 2024 May 21;10:107. doi: 10.1038/s41531-024-00725-y (PMC11109103; doi:10.1038/s41531-024-00725-y)
Supplement: Supplementary file 2 — Reporting summary [file 41531_2024_725_MOESM2_ESM.pdf]

nature portfolio

Corresponding author(s): Gary Ho

Last updated by author(s): Apr 23, 2024

Reporting Summary

Nature Portfolio wishes to improve the reproducibility of the work that we publish. This form provides structure for consistency and transparency in reporting. For further information on Nature Portfolio policies, see our Editorial Policies and the Editorial Policy Checklist.

Statistics

For all statistical analyses, confirm that the following items are present in the figure legend, table legend, main text, or Methods section.

n/a	Confirmed	
☐	☒	The exact sample size (*n*) for each experimental group/condition, given as a discrete number and unit of measurement
☐	☒	A statement on whether measurements were taken from distinct samples or whether the same sample was measured repeatedly
☐	☒	The statistical test(s) used AND whether they are one- or two-sided<br>*Only common tests should be described solely by name; describe more complex techniques in the Methods section.*
☒	☐	A description of all covariates tested
☐	☒	A description of any assumptions or corrections, such as tests of normality and adjustment for multiple comparisons
☐	☒	A full description of the statistical parameters including central tendency (e.g. means) or other basic estimates (e.g. regression coefficient) AND variation (e.g. standard deviation) or associated estimates of uncertainty (e.g. confidence intervals)
☐	☒	For null hypothesis testing, the test statistic (e.g. *F*, *t*, *r*) with confidence intervals, effect sizes, degrees of freedom and *P* value noted<br>*Give P values as exact values whenever suitable.*
☒	☐	For Bayesian analysis, information on the choice of priors and Markov chain Monte Carlo settings
☒	☐	For hierarchical and complex designs, identification of the appropriate level for tests and full reporting of outcomes
☒	☐	Estimates of effect sizes (e.g. Cohen's *d*, Pearson's *r*), indicating how they were calculated

Our web collection on statistics for biologists contains articles on many of the points above.

Software and code

Policy information about availability of computer code

Data collection	All software and codes are commercially or freely available.
Data analysis	Western blot quantifications were performed using LiCor ImageStudio. Statistical analyses done using Graphpad Prism 10. Image analysis in immunofluorescence and sypHy assays performed using the freely available FIJI.

For manuscripts utilizing custom algorithms or software that are central to the research but not yet described in published literature, software must be made available to editors and reviewers. We strongly encourage code deposition in a community repository (e.g. GitHub). See the Nature Portfolio guidelines for submitting code & software for further information.

Data

Policy information about availability of data

All manuscripts must include a data availability statement. This statement should provide the following information, where applicable:

- Accession codes, unique identifiers, or web links for publicly available datasets
- A description of any restrictions on data availability
- For clinical datasets or third party data, please ensure that the statement adheres to our policy

All data generated (and non-commercially available tools used in experiments) are included in this published article and its Supplementary Information section.

Research involving human participants, their data, or biological material

Policy information about studies with human participants or human data. See also policy information about sex, gender (identity/presentation), and sexual orientation and race, ethnicity and racism.

Reporting on sex and gender	N/A; no human participants.
Reporting on race, ethnicity, or other socially relevant groupings	N/A; no human participants.
Population characteristics	N/A; no human participants.
Recruitment	N/A; no human participants.
Ethics oversight	N/A; no human participants.

Note that full information on the approval of the study protocol must also be provided in the manuscript.

Field-specific reporting

Please select the one below that is the best fit for your research. If you are not sure, read the appropriate sections before making your selection.

☒ Life sciences ☐ Behavioural & social sciences ☐ Ecological, evolutionary & environmental sciences

For a reference copy of the document with all sections, see nature.com/documents/nr-reporting-summary-flat.pdf

Life sciences study design

All studies must disclose on these points even when the disclosure is negative.

Sample size	Sample sizes were chosen based on previously determined effect sizes and power analyses.
Data exclusions	No data was excluded.
Replication	All data were reproduced by two individuals conducting the same experiments separately on different days. Neurons used for a given set of data used in each figure in this paper were from at least 2 distinct differentiations.
Randomization	N/A
Blinding	Blinding was not done.

Reporting for specific materials, systems and methods

We require information from authors about some types of materials, experimental systems and methods used in many studies. Here, indicate whether each material, system or method listed is relevant to your study. If you are not sure if a list item applies to your research, read the appropriate section before selecting a response.

Materials & experimental systems

n/a	Involved in the study	
☐	☒	Antibodies
☐	☒	Eukaryotic cell lines
☒	☐	Palaeontology and archaeology
☒	☐	Animals and other organisms
☒	☐	Clinical data
☒	☐	Dual use research of concern
☒	☐	Plants

Methods

n/a	Involved in the study	
☒	☐	ChIP-seq
☒	☐	Flow cytometry
☒	☐	MRI-based neuroimaging

Antibodies

Antibodies used	actin (mouse, Sigma A3853), αS (rabbit, Abcam ab138501, clone MJFR1), beta-3-tubulin (chicken, Abcam ab41489), GAPDH (rabbit, Abcam ab181602), Homer1 (rabbit, synaptic systems 160003), N-cadherin (rabbit, Abcam ab76011), PMCA1 (rabbit, Abcam ab190355), POU3F2/Brn2 (rabbit, CST mab#12137), SNAP-25 (rabbit, Abcam ab5666), vGlut2 (mouse, Abcam ab79157 clone 8G9.2).

Validation	Antibodies are all commercially available and validated by the manufacturer.

Eukaryotic cell lines

Policy information about cell lines and Sex and Gender in Research

Cell line source(s)	BR33 and 2132 lines are available through the associated references provided in the methods section.
Authentication	Authentication performed through our in-house iPSC neurohub core.
Mycoplasma contamination	Cells were regularly tested for mycoplasma contamination using the Sigma LookOut PCR mycoplasma detection kit
Commonly misidentified lines (See ICLAC register)	None

Plants

Seed stocks	*Report on the source of all seed stocks or other plant material used. If applicable, state the seed stock centre and catalogue number. If plant specimens were collected from the field, describe the collection location, date and sampling procedures.*
Novel plant genotypes	*Describe the methods by which all novel plant genotypes were produced. This includes those generated by transgenic approaches, gene editing, chemical/radiation-based mutagenesis and hybridization. For transgenic lines, describe the transformation method, the number of independent lines analyzed and the generation upon which experiments were performed. For gene-edited lines, describe the editor used, the endogenous sequence targeted for editing, the targeting guide RNA sequence (if applicable) and how the editor was applied.*
Authentication	*Describe any authentication procedures for each seed stock used or novel genotype generated. Describe any experiments used to assess the effect of a mutation and, where applicable, how potential secondary effects (e.g. second site T-DNA insertions, mosiacism, off-target gene editing) were examined.*
